# Immunogenicity and protective efficacy of a *Streptococcus suis* vaccine composed of six conserved immunogens

**DOI:** 10.1186/s13567-021-00981-3

**Published:** 2021-08-25

**Authors:** Christine Weiße, Denise Dittmar, Beata Jakóbczak, Volker Florian, Nicole Schütze, Gottfried Alber, Kristin Klose, Stephan Michalik, Peter Valentin-Weigand, Uwe Völker, Christoph Georg Baums

**Affiliations:** 1 Institute of Bacteriology and Mycology, Centre for Infectious Diseases, Faculty of Veterinary Medicine, Leipzig University, Leipzig, Germany; 2 Department of Functional Genomics, Interfaculty Institute for Genetics and Functional Genomics, University Medicine Greifswald, Greifswald, Germany; 3 Ceva Innovation Center GmbH, Dessau-Rosslau, Germany; 4 Institute of Immunology, Centre for Infectious Diseases, Faculty of Veterinary Medicine, Leipzig University, Leipzig, Germany; 5 Institute of Pathology, Faculty of Veterinary Medicine, Leipzig University, Leipzig, Germany; 6 Department of Infectious Diseases, Institute for Microbiology, University of Veterinary Medicine Hannover, Hannover, Germany

**Keywords:** TroA, OppA, Basic membrane lipoprotein, LysM, Di-peptidyl peptidase IV, Subtilisin-like serine protease, Nucleoside ABC transporter, Bactericidal assay, Immunogens

## Abstract

A vaccine protecting against different *Streptococcus suis* serotypes is highly needed in porcine practice to improve animal welfare and reduce the use of antibiotics. We hypothesized that immunogens prominently recognized by convalescence sera but significantly less so by sera of susceptible piglets are putative protective antigens. Accordingly, we investigated immunogenicity and protective efficacy of a multicomponent vaccine including six main conserved immunogens, namely SSU0934, SSU1869, SSU0757, SSU1950, SSU1664 and SSU0187. Flow cytometry confirmed surface expression of all six immunogens in *S. suis* serotypes 2, 9 and 14. Although prime-booster vaccination after weaning resulted in significantly higher specific IgG levels against all six immunogens compared to the placebo-treated group, no significant differences between bacterial survival in blood from either vaccinated or control animals were recorded for serotype 2, 9 and 14 strains. Furthermore, vaccinated piglets were not protected against morbidity elicited through intranasal challenge with *S. suis* serotype 14. As ~50% of animals in both groups did not develop disease, we investigated putative other correlates of protection. Induction of reactive oxygen species (ROS) in blood granulocytes was not associated with vaccination but correlated with protection as all piglets with >5% ROS survived the challenge. Based on these findings we discuss that the main immunogens of *S. suis* might actually not be a priori good candidates for protective antigens. On the contrary, expression of immunogens that evoke antibodies that do not mediate killing of this pathogen might constitute an evolutionary advantage conserved in many different *S. suis* strains.

**Supplementary Information:**

The online version contains supplementary material available at 10.1186/s13567-021-00981-3.

## Introduction

*Streptococcus suis* is a very successful colonizer of mucosal surfaces in pigs. However, it is also a major porcine pathogen causing severe pathologies such as meningitis, polyarthritis, septicemia and endocarditis. Currently, 29 serotypes (*cps*) have been confirmed [1]. Strains of *cps1*, *cps2*, *cps1/2, cps3*, *cps4*, *cps7*, *cps9* and *cps14* are associated with diseases and main herd problems in different countries, whereas other serotypes contribute only marginally to morbidity [2]. Worldwide, *S. suis cps2* is most frequently isolated from clinical cases in pigs and humans [2]. However, *cps9* has become most prevalent among invasive isolates in some European countries with a large pig industry such as The Netherlands and Spain [2, 3]. In South America *cps14* ranks third among invasive *S. suis* strains [4, 5]. Furthermore, *cps14* is also frequently found in the United Kingdom [3, 6] and contributes substantially to severe zoonotic cases in Asia [2].

As no licensed vaccine has convincingly reduced the burden of *S. suis* diseases in the field, different vaccination approaches are currently under investigation. This includes conjugated capsule polysaccharides [7], live attenuated *Salmonella enterica* serovar Choleraesuis vectors delivering conserved surface proteins [8, 9] and different recombinant antigens [10, 11]. A multicomponent vaccine including five recombinant antigens, which were identified in a screen for fitness genes important for colonization of the pig nasal epithelium, elicits partial protection against a homologous *cps2* challenge [12]. Various groups have been using immunoproteomics to identify conserved immunogens, which are discussed as promising protective antigens. Main immunogens of virulent *cps2* strains include lipoproteins, muramidase-released protein, surface antigen one and suilysin [13]. However, data demonstrating protective efficacy of these immunogens is very limited or even contradictory.

In this study we investigated the protective efficacy of a multicomponent vaccine against *cps14* in the natural host. The multicomponent vaccine included immunogens expressed by at least three different important *S. suis cps* (2, 14, 9). Sera drawn from convalescent piglets contained significantly higher levels of immunogen-specific IgG antibodies than sera drawn from susceptible piglets. Although significant differences in specific antibody levels were elicited through vaccination, protection was not observed. The heterogeneity in the clinical outcome between the different animals was used to investigate a new putative correlate of protection: the induction of reactive oxygen species (ROS, also described as oxidative burst) in blood granulocytes.

## Materials and methods

### Bacterial strains and growth conditions

*S. suis* strain 10 is an *mrp* + *epf* + *sly* + *cps2*+ strain of sequence type 1 that has been used by different groups successfully to induce disease experimentally [14]. Strains 13-00283-02 (*cps7* + *mrp* +) and 16085/3b (*cps9* + *mrp* + *sly* +) are virulent and highly virulent strains of sequence types 27 and 94, respectively [15, 10]. Strain V3117/2 of *cps14* and sequence type 1 was originally isolated from the brain of a pig with meningitis. Sequencing of the *cps*K gene [16, 17] and serotyping by slide agglutination using specific rabbit antisera (ID-Lelystad, Lelystad, The Netherlands) confirmed that strain V3117/2 is a *cps14* strain. The latter was kindly conducted by Astrid de Greef (Wageningen Bioveterinary Research, Lelystad, The Netherlands). Strain 7119-1 (*mrp* + *cps2*+) of sequence type 28 was isolated from the joint fluid of a piglet, which developed arthritis before the start of the experimental trial described below. Bacteria were cultured on Columbia agar plates supplemented with 6% sheep blood or in BactoTM Todd Hewitt broth (THB) at 37 °C for 24 h or overnight, if not stated otherwise. For the use in bactericidal assays, strains were grown in THB until late exponential phase and stored with 20% (v/v) glycerol at −80 °C. *Escherichia* (*E.*) *coli* was cultured in Luria–Bertani (LB) medium. If appropriate, 100 μg/mL ampicillin was added*.*

### Genotyping

Genotyping of *S. suis* strains isolated from pigs was conducted by MP-PCR detecting *mrp*, *epf*, *sly*, *arcA*, *gdh*, *cps1/14*, *cps2*, *cps7* and *cps9* as well as with an *epf* monoplex variant PCR [18]. Sanger sequencing of the *cps*K locus was conducted to identify *cps14* among *cps1* positive strains [16, 17]. Multi locus sequence typing (MLST) was performed as described previously [15].

### Expression and purification of recombinant (r) proteins

To express recombinant SSU0934, SSU1869, SSU0757, SSU1950, SSU1664, SSU0309 and SSU0187 in *E. coli,* plasmids were constructed as follows. Genomic DNA of *S. suis* (strain 10, *cps*2) was used to amplify genes by PCR. The DNA fragments of *ssu0934*, *ssu1869*, *ssu0757*, *ssu1950*, *ssu1664* and *ssu0187* were generated using 0934(-21aa)-F/0934-R, 1869(-20aa)-F/1869-R, 757Pro-F/757Pro-R, 1950(-30aa)-F/1950-R, 1664(-31aa)-F/1664-R and 0187-F/0187(-stop)-R primer pairs, respectively (Primer sequences are listed in Additional file 1). The products of *ssu0934* and *ssu1950* were digested with *Kpn*I and *Hind*III. The fragments of *ssu1869* and *ssu1664* were digested with *Sac*I and *Sal*I, whereas the products of *ssu0757* and *ssu0187* were digested with *Sal*I and *Bam*HI or *Hind*III and *Sac*I, respectively. The gene products of *ssu0934*, *ssu1869*, *ssu0757*, *ssu1950* and *ssu1664* were cloned into pQE80L vector (Qiagen, Hilden, Germany) or pQE80L-Strep vector for *ssu0187* digested with the appropriate enzymes. pQE80L-Strep vector was constructed by introducing Strep-tag sequence C-terminal into the MCS of the pQE80L vector by PCR using primers pQE80L-strep-F/pQE80L-HindIII-R.

The DNA fragment of *ssu0309* was synthesized and cloned into a standard vector at the restriction sites of *Sac*I and *Bam*HI by the company Eurofins Genomics GmbH. The gene was subcloned into pQE80L vector using *Sac*I and *Bam*HI enzymes. The cloned sequence encodes the protein sequence of SSU0309 (GenBank: CAR44738.1) from amino acid 20 to 1051.

Plasmid constructs were transformed to *E. coli* TOP10F’ cells (Invitrogen, Thermo Fisher Scientific, Dreieich, Germany). The purified plasmid DNA was sequenced for verification (conducted by Eurofins MWG Operon, Ebersberg, Germany).

*E. coli* cultures were grown shaking in 400 mL LB medium at 30 °C to an OD_600nm_ of 0.5–0.7. Four-hour overexpression of proteins was induced by addition of IPTG (dioxane-free, Thermo Fisher Scientific, Dreieich, Germany) at a final concentration of 0.1 mM (SSU1869, SSU0757, SSU1950, SSU0309 and SSU0187) or 1 mM (SSU0934 and SSU1664). The cells were harvested by centrifugation at 9150 × *g* for 10 min at room temperature and resuspended in LEW buffer (50 mM NaH_2_PO_4_, 300 mM NaCl, pH 8.0) to the final OD_600nm_ of 60. To purify SSU0934, SSU1869 and SSU0757 cells were disrupted by sonication and centrifuged at 26 900 × *g* for 30 min at 4 °C to dispose cell debris. The resulting supernatant was used for the protein purification. Proteins were purified under native conditions using Ni-IDA 2000 packed columns (Protino, Macherey–Nagel, Düren, Germany) as recommended by the manufacturer. The column was washed twice with LEW buffer containing 20 mM imidazole. Proteins were eluted with elution buffer (50 mM NaH_2_PO_4_, 300 mM NaCl, 250 mM imidazole, pH 8.0). The purified proteins were dialyzed against dialysis buffer (300 mM NaCl, 9.3 mM Na_2_HPO_4_, 1.6 mM NaH_2_PO_4_, pH 7.4).

To purify SSU0309 cell suspension was incubated for 20 min at room temperature after addition of lysozyme (from chicken egg white, Sigma-Aldrich, Taufkirchen, Germany) and protease inhibitor cocktail (VWR, Darmstadt, Germany) to the final concentration of 1 mg/mL and 50 µL/1 g pellet, respectively. Cells were disrupted by sonication and centrifuged at 26 900 × *g* for 15 min at 4 °C to dispose cell debris. The protein was purified as described for SSU0934, SSU1869 and SSU0757 with some alterations, namely, the column was washed twice with LEW buffer and the protein was dialyzed against PBS buffer (146 mM NaCl, 9.3 mM Na_2_HPO_4_, 1.6 mM NaH_2_PO_4_).

To purify SSU1950 and SSU1664, cells were disrupted by addition of BugBuster (10 × concentrated, protein extraction reagent, EMD Millipore, Merck KGaA, Darmstadt, Germany), lysozyme and Benzonase (EMD Millipore) to the final concentration of one time concentrated, 1 mg/mL and 12.5 U/mL, respectively. The mixtures were incubated for 20 min at room temperature and further centrifuged at 26 900 × *g* for 30 min at 20 °C. Proteins were purified as described for SSU0934, SSU1869 and SSU0757. To purify SSU0187, cells were disrupted by sonication and processed as described for SSU0934, SSU1869 and SSU0757. SSU0187 was purified using Strep-Tactin resin (Superflow Plus from Qiagen). The supernatant was incubated with appropriate amounts of the beads with gentle shaking at 4 °C for 1 h. The mixture was loaded on a laboratory column (with 10 µm filter pore size, MoBiTec, Göttingen, Germany). The resin was washed six times with LEW buffer. The proteins were eluted with LEW buffer enriched with 2.5 mM d-desthiobiotin (Novagen, Merck KGaA, Darmstadt, Germany). The purified protein was dialyzed as described for SSU0934, SSU1869 and SSU0757. Purity of all proteins was assessed by SDS-PAGE and validated by mass spectrometric analysis.

### MS sample preparation and measurement

For analysis of the recombinant proteins by mass spectrometry, 2 µg of total protein was prepared with the single pot solid-phase enhanced sample preparation (SP3) protocol [19]. Liquid chromatography electro spray ionization tandem mass spectrometry (MS/MS) on a LTQ Orbitrab Velos instrument selecting TOP 20 precursor ions for CID fragmentation per cycle was performed as described earlier [20]. Data analysis was carried out using MaxQuant 1.5.3.8 [21]. Peptides were identified by search against the Uniprot *E. coli* database (release 08/2017) spiked with the sequences of the recombinant proteins. The following settings were used: Trypsin/P as proteolytic enzyme, two missed cleavage and methionine oxidation. Only peptides identified with a PSM false discovery rate (FDR) 0.01 were used for further analysis. Proteins were only identified, if two or more unique peptides were found per protein. The mass spectrometry analysis allowed assignment of the signal intensities predominantly to the recombinant proteins: SSU0187 ≥ 97%, SSU0934 = 91%, SSU1664 = 91%, SSU1869 = 90%, SSU0757 = 88%, and SSU1950 = 84%. The residual intensity was distributed among various *E. coli* proteins.

### Classification of sera used for characterization of immunogens

Sera used for characterization of immunogens originated from various experimental infections with different *S. suis* strains or from bacterin vaccination as specified in Additional files 2 and 3, respectively. The protocols for the animal experiments were either approved by the Committee on Animal Experiments of the Lower Saxonian State Office for Consumer Protection and Food Safety (Niedersächsisches Landesamt für Verbraucherschutz und Lebensmittelsicherheit, LAVES under the permit number 509.6-42502-04/829; 33.14-42502-04-12/0965; 33.12-42502-04-16/2305A) or the Saxony Regional Office (Landesdirektion Sachsen under the permit no. TVV14/15; TVV26/15; N01/16; TVV11/16; TVV28/16; TVV37/17). All studies were performed in strict accordance with the principles and recommendations outlined in the European Convention for the Protection of Vertebrate Animals Used for Experimental and Other Scientific Purposes (European Treaty Series, no. 123) and the German Animal Protection Law (Tierschutzgesetz). The porcine sera used to characterize the immunogens were classified as follows:

“Sera pre-infection” (*n* = 20) were defined as sera drawn prior to experimental *S. suis* infection of piglets surviving the following challenge to the end of the observation period of at least two weeks. Sera collected at the end of this observation period are in the group “Sera post-infection”.

“Sera post-infection” (*n* = 20) were defined as sera drawn at the end of the observation period after experimental *S. suis* infection (also referred to as sera of reconvalescent pigs). The length of the observation period ranged from 14 to 22 days. At the point of serum collection neither clinical nor bacteriological nor histological examinations revealed any signs of an acute *S. suis* infection. *S. suis* challenge strains in these experiments belonged either to *cps2* (*n* = 10), *cps9* (*n* = 3), *cps7* (*n* = 5) or *cps14* (*n* = 2) (see Additional file 2).

“Sera of susceptible piglets” (*n* = 20) were defined as sera drawn prior to an experimental *S. suis* infection that resulted in a fatal *S. suis* disease in this piglet (also including piglets euthanized due to reaching termination criteria). Pathologies in these piglets included meningitis, serositis and polyarthritis as confirmed by pathohistological investigations in association with detection of the *S. suis* challenge strain of either *cps2* (*n* = 8), *cps9* (*n* = 5), *cps7* (*n* = 4) and *cps14* (*n* = 3) (see Additional file 2).

“Hyperimmune sera” (*n* = 16) were defined as sera drawn from prime-booster vaccinated pigs not experimentally infected with *S. suis*. Vaccination was conducted either with a *cps2* (*n* = 8), a *cps7* (*n* = 2) or a *cps9* (*n* = 6) bacterin (see Additional file 3).

### Determination of IgG antibody titers using bead-based xMAP^®^ technology

The IgG antibody titers of 76 porcine sera (Additional files 2, 3) against recombinant purified *S. suis* proteins were determined using bead-based xMAP^®^ technology (Luminex^®^) [22]. The analysis of the purified Strep- or His-tagged proteins was performed according to a modified method from Meyer et al. [23]. For detection of antigen-specific IgG a series of seven serum dilutions was investigated (50-fold, 250-fold, 1000-fold, 5000-fold, 50 000-fold, 100 000-fold and 200 000-fold). The amount of swine IgG antibodies bound to each antigen was detected via a goat anti-porcine IgG antibody (H + L) conjugated with PE (R-phycoerythrin) (6050-09, SouthernBiotech, Birmingham, USA). The data analysis was performed using the xMAPr app [23].

### Generation of rabbit anti-sera

Immunizations of rabbits with recombinant variants of SSU1869, SSU0757, SSU1950 and SSU0187 and respective blood collections were performed by *DAVIDS Biotechnologie GmbH* (Regensburg, Germany). Generation of hyperimmune sera in rabbits against SSU0934 and SSU1664 was conducted by ourselves as described [24] and was registered at the LAVES under 87A044.

### SDS-PAGE and Western blot analysis

SDS-Page and Western blot analysis was performed essentially as described previously [24]. Rabbit sera against proteins of interest were used in a dilution of 1:5000. The secondary antibody, goat-anti-rabbit IgG HRP (catalogue 111-035008, Dianova, Hamburg, Germany), was diluted 1:25 000.

### Detection of antigens on the surface of *S. suis* through flow cytometry

For flow cytometry analysis, bacteria were grown in THB or porcine plasma until early stationary phase and stored in 20% (v/v) glycerol at −80 °C. Plasma was collected from piglets at the age of 7–9 weeks from a herd that is known to be infected with several *S. suis* serotypes. The collection of blood samples was approved by the Landesdirektion Sachsen (permit no. TVV40/18). Before use, plasma samples were pooled. Bacteria-glycerol solutions containing 6.5 × 10^6^ CFU were centrifuged at 4 °C for 10 min at 2100 × *g*, supernatant was discarded and pellets were washed twice with phosphate buffered saline (PBS) to remove remaining glycerol. Pellets were blocked with 300 µL 1:100 diluted normal donkey serum (Dianova, catalogue #017-000-121) for 30 min at 4 °C on the rotator, followed by staining with primary and secondary antibodies for 30 min at 4 °C rotating as well. Sera from rabbits immunized with one of the recombinant antigens of the multicomponent vaccine (1:50 dilution in PBS) served as primary antibodies. Serum of a non-immunized rabbit was used as the control. As secondary antibody we used a 1:200 diluted fluorescein isothiocyanate (FITC)-labeled donkey anti-rabbit IgG (Dianova, catalogue #DAB-87179). Between every incubation step, pellets were washed twice with PBS. Samples were fixated with 2% (w/v) paraformaldehyde. Flow cytometry measurement was conducted with BD, FACS Fortessa and data were analyzed using the FlowJoTM_V10 software.

### Animal experiments

Using a litter-matched design, 18 German landrace piglets from a herd known to be free of *cps1*, *cps7*, *cps9*, *cps14* but not *cps2* were divided into two groups (placebo: *n* = 9; vaccinated: *n* = 9). The classification of the herd was based on the MP-PCR typing results [18] of *S. suis* isolates from the tonsils of more than 500 animals over the last 16 years [in the following these piglets are referred to as specific pathogen free (spf)]. Piglets were prime-booster immunized intramuscularly at the age of four and six weeks either with the multicomponent vaccine or a placebo with PBS, both supplemented with 20% (v/v) Emulsigen as adjuvant. Every vaccine dose contained 150 µg of each recombinant immunogen (SSU0934, SSU1869, SSU0757, SSU1950, SSU1664, SSU0187). After weaning, one piglet developed acute disease. This animal and the littermate were excluded from the experiment. Two weeks after booster immunization the remaining 16 animals were infected intranasally with 5 × 10^9^ CFU of *S. suis cps14* V3117/2 grown in Bacto™ Tryptic Soy Broth without dextrose (BD, Heidelberg, Germany). Monitoring of clinical parameters after infection was performed every 8 h including measurement of the inner body temperature, assessment of movements and feed intake using a score sheet as described previously [10] with the following modification: moderate and ceased feed intake received scores of 2 and 5, respectively. Piglets were only fed at these time points. Piglets exhibiting high fever (≥ 40.5 °C) combined with apathy and anorexia (over 24 h), as well as animals showing any clinical signs of acute polyarthritis or severe meningitis, were euthanized for reasons of animal welfare. Surviving piglets were sacrificed fourteen days post-infection. All animals went through the same necropsy, histopathological and bacteriological screenings as described previously [10].

Animals were infected experimentally and cared for in accordance with the principles outlined in the EU Directive 2010/63/EU. All animal experiments or samplings were conducted by veterinarians and in accordance with the principles outlined in the European Convention for the Protection of Vertebrate Animals Used for Experimental and other Scientific Purposes and the German Animal Protection Law (Tierschutzgesetz). The animal experiment of this study was approved by the Saxony Regional Office (permit no. TVV57/18).

### Bactericidal assay

Prior immunization and eleven days after booster, heparinized blood was collected from all animals and a bactericidal assay was conducted. Five hundred microliters of heparinized blood (16 I. U. heparin/mL) were mixed with either 1.2 × 10^6^ CFU of *S. suis* strain 10 or 6 × 10^6^ CFU of 16085/3b or 3 × 10^6^ CFU of V3117/2 and incubated for 2 h at 37 °C on a rotator. Before and after incubation, CFU were determined by plating of serial dilutions. Survival factors represent the ratio of CFU at 120 min to CFU at timepoint zero. Survival factors > 1.0 show proliferation, whereas survival factors < 1.0 indicate killing of *S. suis.*

### Opsonophagocytosis assay

Blood was collected from weaning piglets from a herd which is known to be infected with several *S. suis cps* (permit no. N19/14 and A09/19). Porcine neutrophils were separated using density centrifugation as described before [25]. Purified porcine neutrophils (5 × 10^6^ in 400 µL RPMI) were mixed with 100 µL of porcine serum and 3 × 10^5^ CFU of *S. suis* strains 10 or 13-00283-02 or 16085/3b and incubated for 1 h at 37 °C on a rotator. We used serum of colostrum-deprived piglets as the negative control. Hyperimmune serum from an animal vaccinated with a *cps2* bacterin served as the positive control. CFU were determined before and after incubation and survival factors were calculated as mentioned before for the bactericidal assay.

### Reconstituted blood assay with pre-adsorbed sera

The *cps2 S. suis* isolate 7119-1 of a piglet, which developed severe disease before experimental infection, was used to pre-adsorb sera from animals collected eleven days post booster immunization. For each sample, 5 mL of overnight culture in THB was centrifuged for 15 min at 6620 × *g*, supernatant was discarded and the pellets were washed twice with PBS. Bacteria were resuspended in 500 µL piglet sera and incubated for 1 h rotating at 4 °C. Afterwards, samples were centrifuged for 20 min at 10 000 × *g* and the supernatant transferred to a 0.22 µm UltrafreeMC centrifugal filter (UFC30GV0S, Merck Millipore, Merck KGaA, Darmstadt, Germany) and centrifuged for 4 min at 12 000 × *g*. Sera were used to reconstitute blood for bactericidal assays as follows. Heparinized blood of a healthy pig (11^th^ week, permit no. A09/19) was centrifuged at 500 × *g* for 10 min. Plasma was removed and blood cells were washed twice with PBS and mixed 1:1 (v/v) with 100 µL of pre-adsorbed serum or untreated control serum and 3 × 10^5^ CFU/mL of *cps14* strain V3117/2. CFU were determined before and after an incubation of 2 h at 37 °C on a rotator. Survival factors were calculated as mentioned before.

### Detection of *S. suis* induced oxidative burst in granulocytes

Oxidative burst was detected as previously described [26]. Briefly, *S. suis cps14* strain V3117/2 was added at a concentration of 6 × 10^6^ CFU/mL to heparinized blood samples. To determine *S. suis* induced signals, for each blood sample a PBS control (instead of *S. suis*) was used. As a positive control 0.1 µg/mL PMA (Sigma-Aldrich) stimulation was used instead of *S. suis*. Detection of phagocytosis in combination with oxidative burst was done with *cps2*-preadsorbed sera and untreated control sera in a reconstituted assay using heparinized blood of a healthy piglet (11^th^ week permit no. A09/19). To remove the plasma, the blood was washed twice with PBS and was finally adjusted to three-quarter original volume with PBS. *S. suis*, V3117/2, prestained with Cell Trace FarRed (Thermo Fisher Scientific), was added and intensively mixed before addition to the test-sera to reach finally 6 × 10^6^ CFU/mL. All samples were incubated at 37 °C for 15 min in a water bath. Subsequently, dihydrorhodamine 123 (Sigma-Aldrich, DHR123; 5 µg/mL) was added and incubation was continued for another 10 min at 37 °C. Finally, erythrocytes were lysed two times using erythrocyte lysis buffer (0.155 M ammonium chloride, 10 mM potassium bicarbonate, 0.1 mM disodium EDTA, pH 7.2). The remaining leucocytes were washed two times with PBS and finally samples were fixed with 2% (w/v) paraformaldehyde and measured immediately by flow cytometry (BD FACSCalibur). Data analysis was conducted by gating on granulocytes (FlowJo). *S. suis*-induced oxidative burst was defined as the difference: % Rhodamine123 (Rho123) + granulocytes in *S. suis* sample − % Rho123 + granulocytes in PBS control.

### ELISA

IgM and IgG antibody levels of pre-adsorbed and control sera were determined following a standard protocol as described before, but with minor modifications [15]. Briefly, Nunc Immuno MaxiSorp plates (SigmaAldrich) were coated overnight at 4 °C with 0.2% (v/v) formaldehyde-inactivated bacteria of strain V3117/2. Convalescent sera of five piglets, experimentally infected with V3117/2, were mixed in equal proportions and used as reference serum to define 100 ELISA units. Plates were blocked and dilutions were made in PBS with 0.5% (w/v) bovine serum albumin (BSA) and 1% (w/v) gelatin. For IgM detection a polyclonal secondary goat-anti-porcine-IgM horseradish-peroxidase (HRP) conjugated antibody (NBP2-42699H, 1:10 000, Novus Biologicals, Wiesbaden-Nordenstadt, Germany) and for IgG detection a polyclonal goat anti-porcine IgG HRP conjugated antibody (A100-105P, 1:10 000, Bethyl, Hamburg, Germany) was used (1 h incubation time each).

### Statistical analysis

IgG titers of the different groups were compared using a Wilcoxon rank sum test. Multiple test adjusted (Benjamini–Hochberg procedure) [27] *p*-values (q-values) below 0.05 were considered statistically significant. All calculations and plot generation were performed in R (v 3.6.1) [28] using the packages: tidyverse (v 1.3.0) [29], helfeRlein (v 0.2.2) [30], plotly (v 4.9.2.1) [31], ggsignif (v 0.6.0) [32], ggplot2 (v 3.3.2) [33], grid (v 3.6.1) [28], gridExtra (v 2.3) [34], FactoMineR (v 2.3) [35], factoextra (v 1.0.7) [36], ggrepel (v 0.8.2) [37], and patchwork (v 1.0.1) [38].

Data of the bactericidal assay was analyzed with the Mann–Whitney-U test for comparison of the vaccinated versus placebo-treated group. The correlation factor between blood survival factors and *S. suis*-induced oxidative burst rates was calculated with Spearman rank correlation. Significant differences between pre-adsorbed and control sera in reconstituted blood assay and IgG-ELISA were determined using the Wilcoxon matched pairs test. A paired t-test was used to compare these groups in phagocytosis assay and IgM-ELISA. Probabilities lower than 0.05 were considered significant (*p*-value: ≤ 0.001 = ***; ≤ 0.01 = **; ≤ 0.05 = *; > 0.05 = NS).

## Results

### Selection of putative protective immunogens

**Figure 1:**
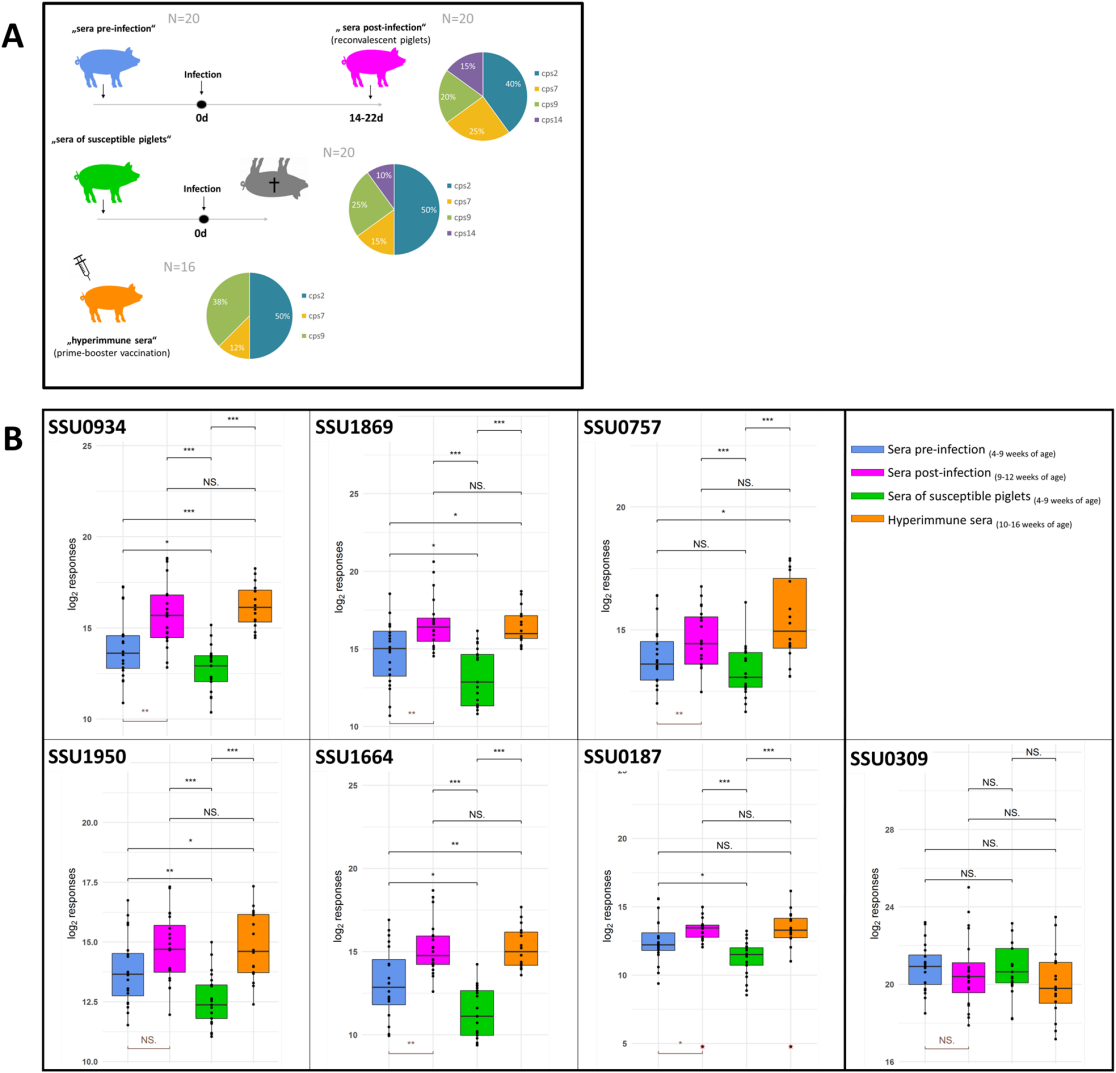
**Classification of porcine sera (A) used to characterize immunogens (B) included in the vaccine.** Sera pre and post-infection were drawn as indicated within the course of experimental *S. suis* infections of non‐vaccinated piglets that survived the experiment to the end of the observation period. Animals that developed severe disease and died or had to be euthanized shortly after infection were classified as susceptible. Sera of these animals were collected before infection. Hyperimmune sera were taken from animals prime-booster vaccinated with a *S. suis* bacterin. The pie charts indicate the proportion of the indicated *S. suis cps* among the experimental infections used for collection of serum samples (**A**). Recognition of *S. suis* proteins SSU0934, SSU1869, SSU0757, SSU1950, SSU1664, SSU0187 and SSU0309 through specific IgG present in the indicated sera (**B**). Detection of serum IgG bound to the indicated antigens coupled to beads was performed using the xMAPr approach [23]. The antigens included in the vaccine recruited significantly more specific IgG in hyperimmune sera and sera post-infection compared to sera of susceptible pigs. SSU0309 is shown as an example of an antigen that did not show these differences. Unpaired Wilcoxon rank sum test (black), paired Wilcoxon rank sum test (brown) p-value: ≤ 0.001 = ***; ≤ 0.01 = **; ≤ 0.05 = *; > 0.05 = NS.

We hypothesized that protective immunogens expressed by different serotypes should be prominently recognized by sera which were drawn from pigs post-infection and which elicited killing of *S. suis* in bactericidal or opsonophagocytosis assays. Sera drawn from susceptible piglets succumbing after challenge to *S. suis* disease should contain much less IgG antibodies binding to these immunogens. Over the last years we have established a large biobank including sera drawn from piglets in experimental *S. suis* studies with a detailed documentation of clinics, pathologies and bacteriologies. All sera included in the study contained *S. suis* specific antibodies as they were collected from piglets colonized with *S. suis.* Blood and serum of these piglets were investigated in bactericidal and opsonophagocytosis assays with different serotypes (Additional file 2). Sera were assigned to different groups as outlined in the Material and methods and Figure 1A. A collection of proteins encoded by genes conserved in the recently published genomes of strains 10 (*cps*2), 13-00283-02 (*cps*7) and 16085/3b (*cps*9) were expressed recombinantly (Additional file 4) [39]. Measurement of IgG titers revealed that post-infection sera contained significantly higher IgG levels against SSU0934, SSU1869, SSU0757, SSU1950, SSU1664 and SSU0187 than sera drawn from susceptible piglets (see Figure 1B), which prompted us to include these six proteins in the vaccine of this study. However, such significant differences in binding of specific IgG in post-infection sera and sera of susceptible piglets were not recorded for all investigated *S. suis* proteins. As an example, SSU0309, a homologue of the pneumococcal histidine triad protein Pht309 [40], displayed similar IgG binding in the different groups of sera (Figure 1B). Noteworthy, in vitro killing of the four investigated *S. suis* strains (of *cps2*, *7*, *14* and *9*) was more prominent in osponophagocytosis or bactericidal assays using sera or blood of the post-infection group than using sera of susceptible piglets (Additional file 2). Differences in specific IgG binding between sera drawn post- and pre-infection, which were significant for all of the six antigens but SSU1950, indicated that the selected *S. suis* antigens are expressed in vivo. Of note, hyperimmune sera drawn from piglets vaccinated with bacterins also contained significantly higher IgG levels against each of the six mentioned immunogens in comparison to sera drawn from susceptible piglets. As outlined in Additional file 3, at least 6 of the 16 hyperimmune sera mediated killing of more than one *cps* in opsonophagocytosis assays. Based on these findings we considered these immunogens putative protective antigens.

### Analysis of expression of selected antigens on the surface of *cps2*, *cps14* and *cps9*

**Figure 2:**
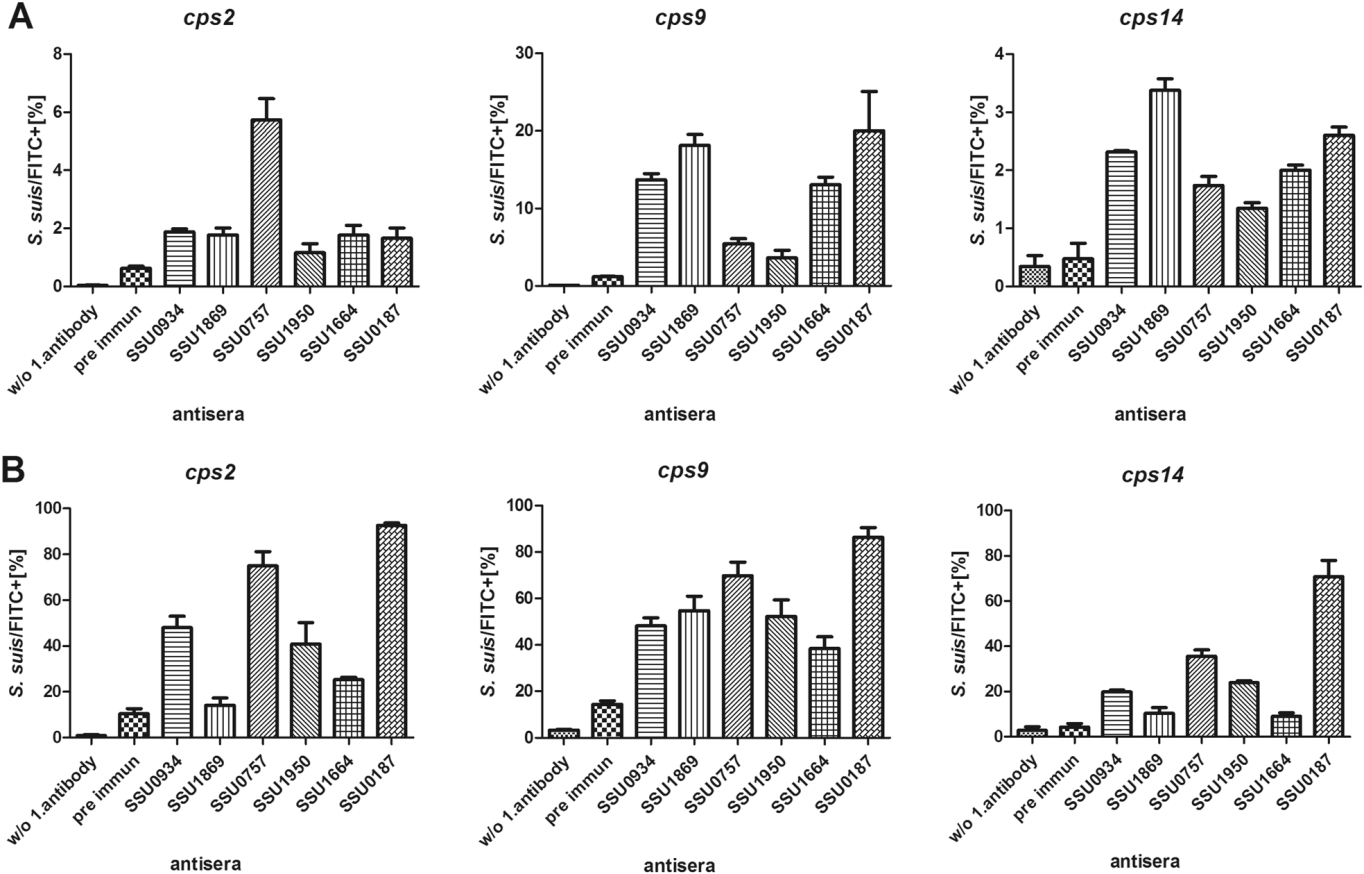
**Detection of vaccine antigens on the bacterial surface after cultivation in THB (A) or porcine plasma** (**B**). *S. suis* strains 10 (*cps2*), 16085/3b (*cps9*) and V3117/2 (*cps14*) were grown until stationary phase and incubated with sera of rabbits immunized with the indicated vaccine antigens. In the next step bacteria were labeled with a fluorescein isothiocyanate (FITC)-labeled anti-rabbit antibody. After fixation with 2% (w/v) paraformaldehyde bacteria were investigated by flow cytometry using a LSR Fortessa (BD Biosciences). Data were analyzed with FlowJo software 10.1r5 (TreeStar, Ashland, OR, USA). As controls, bacteria were measured without incubation in a rabbit antiserum (w/o 1. antibody) or with a rabbit serum collected prior immunization (pre immun). The experiment was repeated three times.

In silico analysis revealed that SSU0934, SSU1869, SSU0757, SSU1950, SSU1664 and SSU0187 are likely secreted or located on the bacterial surface (Table 1). SSU1869 is identical with TroA, a virulence-associated lipoprotein that is necessary for manganese uptake [41]. SSU0757, a virulence-associated subtilisin–like protease on the cell surface of *S. suis*, is described to be able to degrade the Aα chain of fibrinogen and to trigger pro-inflammatory response in macrophages [42]. In strain 16,085/3b an early stop codon within an insertion element results in a truncated ORF (SSU16085_00675) encoding a protein of only 40 kDa without an LPXTG motif [39]. The insertion element carries an IS630 transposase family gene (SSU16085_00676). The 140 kDa large C-terminus of SSU0757 (as annotated in P1/7) might also be expressed in 16085/3b, if UUG is used as an alternative start codon (SSU16085_00677). However, this ORF lacks a signal sequence but carries a C-terminal LPXTG motif (Additional file 4). SSU1950, also known as LysM, is a peptidoglycan-binding protein described to contribute to protection against phagocytosis of a microglia cell line [43]. Putative functions of the other proteins are specified in Table 1. We investigated expression of these proteins on the surface of different serotypes by flow cytometry. This was conducted with each of the rabbit hyperimmune sera elicited through immunization with individual immunogens. In advance, we verified that the hyperimmune sera recognize the recombinant proteins using Western blot analysis (Additional file 5). As shown in Figure 2, flow cytometry suggests that all six immunogens were detectable on the surface of *cps2* strain 10, *cps9* strain 16085/3b and *cps14* V3117/2 grown until early stationary growth in THB or porcine plasma, though antigen-specific labelling varied between strains and also between cultivation in THB and porcine plasma.

**Table 1:** **Immunogens selected for the multicomponent vaccine**

SSU in P1/7	Putative function/ortholog	Localisation/tagg	PSORTb (score)	Theoretical molecular weight (kDa)^a^	Reference
SSU0934	Basic membrane lipoprotein	Signal sequence^b^ Lipobox (LAAC)	Unknown	36	[52]
SSU1869	Zinc ABC transporter TroA	signal sequence^b^	Membrane (9.68)	34	[41, 58]
SSU0757	Subtilisin-like serine protease	signal sequence^b^ LPXTG	Cell wall (10)	168^c^	[42, 59, 60]
SSU1950	LysM protein	signal sequence^b^	Unknown	19	[43, 61]
SSU1664	Oligopeptide substrate-binding protein OppA	Signal sequence^b^ Lipobox (LAAC)	Unknown	64	[44, 45]
SSU0187	Di-peptidyl peptidase IV	No signal sequence^b^	Extracellular (9.6)	88	[62, 63]

^a^Excluding posttranslational modifications.

^b^Detection by SignalP3.0 (using neural networks (NN) and hidden Markov models (HMM) trained on Gram-positive bacteria).

^c^In strain 16085/3b an early stop codon within an insertion results in an ORF of only 40 kDa and without an LPXTG motif.

### Immunogenicities of the multicomponent vaccine

**Figure 3:**
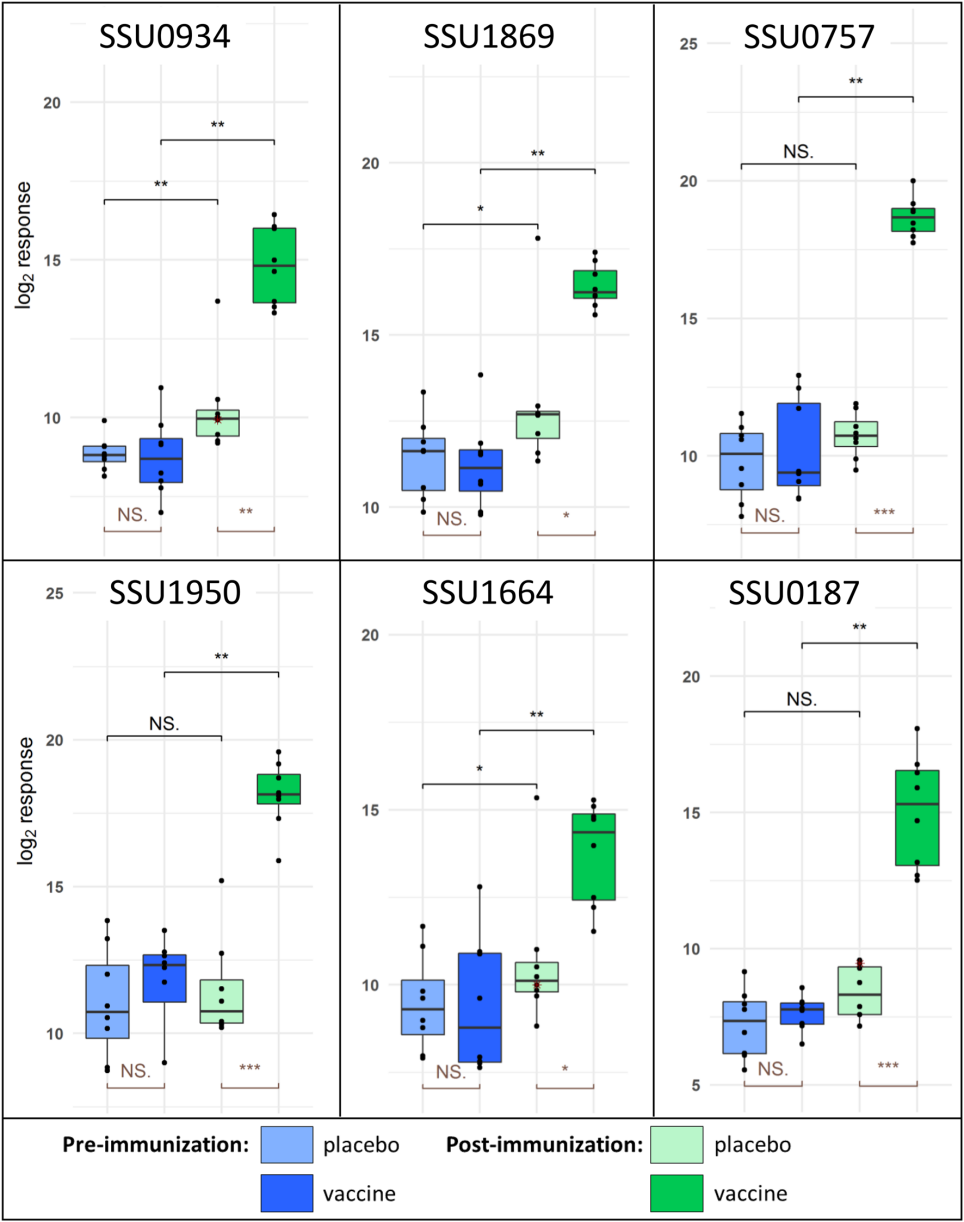
**Immunization elicited significant higher titers of IgG against all six immunogens included in the vaccine.** Serum was collected before prime immunization and eleven days after booster immunization with a multicomponent vaccine (*n* = 8) or a placebo (*n* = 8). Each serum was incubated with beads coupled with selected antigens. Detection of bound serum IgG was performed using the xMAPr approach [23]. Paired Wilcoxon rank sum test (black), unpaired Wilcoxon rank sum test (brown), red star indicates imputed value. *p*-value: ≤ 0.001 = ***; ≤ 0.01 = **; ≤ 0.05 = *; > 0.05 = NS.

**Figure 4:**
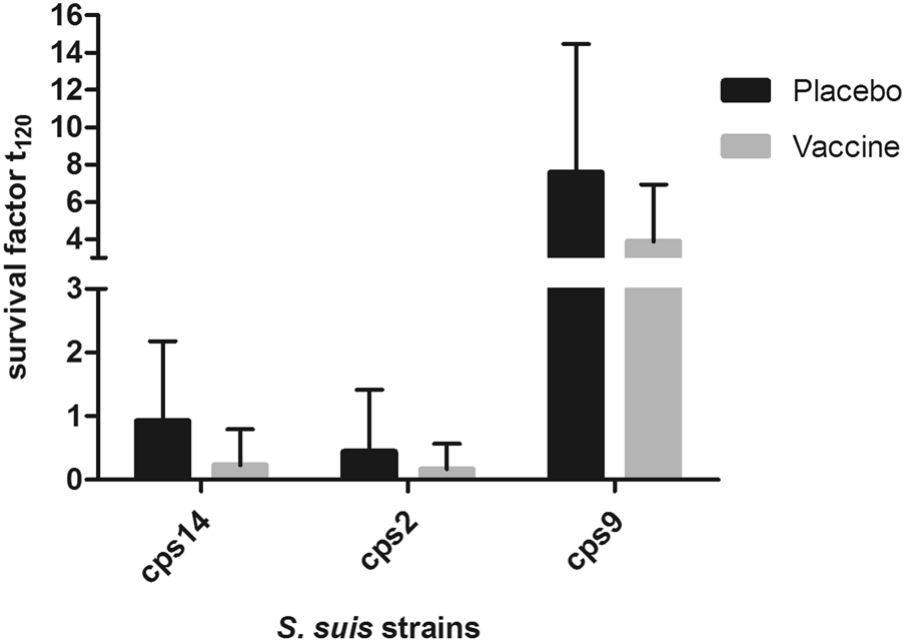
**Survival of *S. suis* in blood drawn from vaccinated or placebo-treated piglets.** Eleven days after booster immunization with a multicomponent vaccine or application of a placebo blood was drawn from 8-week-old piglets (*n* = 8 per group) and incubated with *S. suis* strains V3117/2 (*cps14*), 10 (*cps2*) and 16085/3b (*cps9*). Significant differences were not recorded between vaccinated and placebo-treated piglets in these bactericidal assays using the Mann–Whitney-*U* test. The survival factor represents the ratio of CFU at 120 min to CFU at timepoint zero. Bars and error bars represent mean values and standard deviations, respectively.

Weaning piglets were prime-booster vaccinated with the multicomponent vaccine to investigate immunogenicities and protective efficacies. One piglet developed acute disease related to infection with an *S. suis cps2* strain, designated 7119-1, belonging to sequence type 28 and known to be present in the original herd. It therefore had to be excluded from the experiment together with its littermate. At the start of the experiment, levels of specific IgG antibodies binding to the selected immunogens were rather low in vaccinated and placebo-treated piglets consistent with colonization-induced background antibody levels. After booster immunization the multicomponent group showed significantly higher levels of specific IgG against all six immunogens than before immunization and compared to the control group (Figure 3). We wondered if the induction of systemic IgG levels is associated with reduced proliferation or enhanced killing of different *S. suis* serotypes in blood of these piglets. Accordingly, bactericidal assays were conducted with *S. suis* strains V3117/2, 10 and 16085/3b of *cps14*, *2* and *9*, respectively. Significant differences in the bacterial survival factors were not recorded between placebo-treated and vaccinated piglets eleven days post booster vaccination (Figure 4), although there was a tendency to lower survival factors in the blood of vaccinated piglets for *cps14*, *2* and *9* (mean survival factors of 0.92 ± 1.25 vs. 0.23 ± 0.56 for *cps14*; 0.44 ± 0.97 vs. 0.16 ± 0.40 for *cp*s*2* and 7.56 ± 6.91 vs. 3.87 ± 3.07 for *cps9*, respectively). Survival factors of *cps14* strain V3117/2 were substantially lower than 1 in the vaccinated piglets but much less than in the placebo group, suggesting multicomponent-induced bactericidal activity against *cps14*. These results suggest some limited protection against *cps14*, which prompted us to conduct a *cps14* challenge experiment.

### Protective efficacy of the multicomponent vaccine against *S. suis cps14*

**Figure 5:**
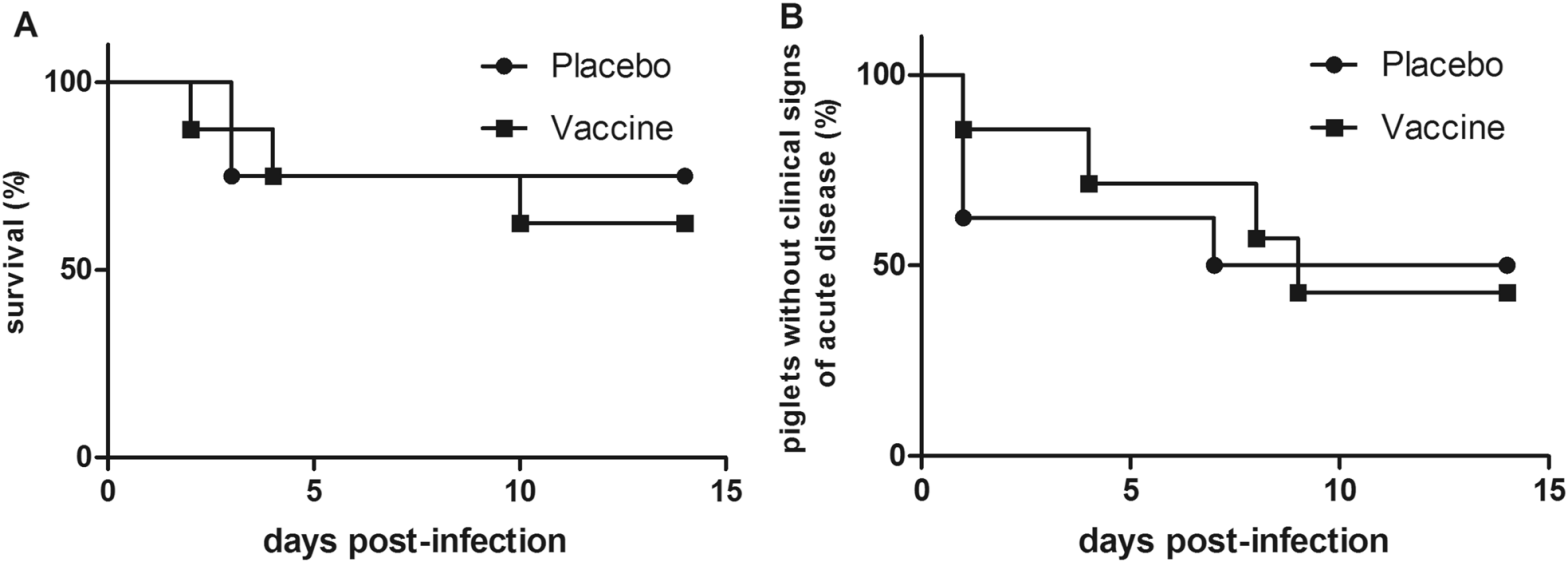
**Mortality (A) and morbidity (B) of vaccinated and placebo-treated piglets induced through *S. suis cps14* infection.** Piglets were challenged intranasally with 5 × 10^9^ CFU of strain V3117/2 (*cps14)* 14 days after booster immunization. Morbidity was defined as piglets showing an inner body temperature of 40.2 °C or higher. In case of high fever (≥ 40.5 °C), apathy and anorexia persisting over 24 h as well as in all cases of central nervous system dysfunction or clinical signs of acute polyarthritis, animals were euthanized for animal welfare reasons. All surviving piglets were sacrificed 14 days post-infection. Significant differences between piglets vaccinated with the multicomponent vaccine (*n* = 8) or the placebo (*n* = 8) were not recorded. Statistical analysis was conducted with the log-rank test.

Vaccinated (*n* = 8) and placebo-treated (*n* = 8) piglets were infected intranasally with *cps14* strain V3117/2 of sequence type 1 fourteen days after booster immunization. Five vaccinated animals and four placebo animals demonstrated clinical signs of severe disease like body temperatures above 40.2 °C or anorexia within the following 10 days (Table 2). In both groups two piglets had to be euthanized for animal welfare reasons because of signs of polyarthritis. In these four early euthanized animals, the challenge strain was detected in multiple inner organs. Furthermore, one vaccinated animal showed additional signs of central nervous system dysfunction (opisthotonus, generalized tremor, ataxia). As shown in Figure 5, significant differences in mortality and morbidity were not recorded between immunized and placebo-treated piglets. Furthermore, detection of the challenge strain in inner organs was very similar in vaccinated and control piglets (Table 3).

**Table 2:** **Evaluation of protection induced by multicomponent vaccine after intranasal challenge with*S. suis cps14* strain V3117/2**

Immunization	Morbidity	Mortality	Mean Clinical score (SD)	Clinical signs				Max. body temperature (°C)		
				CNS^a^	Lameness	No feed intake		< 40	≥ 40 and ≤ 40.2	> 40.2
Placebo	4/8	2/8	7 (11.1)	0/8	2/8	2/8		4/8	0/8	4/8
Vaccine	5/8	3/8	11 (12.0)	1/8	3/8	3/8		3/8	1/8	4/8

^a^Signs of central nervous system (CNS) dysfunction such as convulsions and opisthotonus.

**Table 3:** **Reisolation of the challenge strain from piglets after intranasal challenge with *S. suis cps14* strain V3117/2**

Immunization	Number of piglets positive for the isolation of the challenge strain in an inner organ^b^ or in serosa or in joint fluid	Number of piglets positive for the isolation of the challenge strain in ≥ 3 inner organs^b^	Number of piglets in which the *S. suis* challenge strain^a^ was isolated from							
			Tonsil	Lung^c^	Serosa^d^	Spleen	Liver	Brain, CSF^e^	Joint fluid^f^	Endocard
Placebo	3/8	2/8	4/8	2/8	1/8	1/8	1/8	2/8	2/8	1/8
Vaccine	3/8	3/8	6/8	1/8	0/8	2/8	1/8	2/8	3/8	2/8

^a^The challenge strain was identified by PCR of all isolates and Sanger sequencing of *cps*K locus of five representative isolates from animals showing disseminated infections.

^b^Inner organ refers to lung, spleen, liver, brain, CSF or endocard but not the tonsils.

^c^One cranial lobe was investigated.

^d^Pleural, peritoneal or pericardial cavity.

^e^Cerebrospinal fluid.

^f^Punctures of both tarsal and carpal joints were investigated in each animal. In case of lameness additional joint punctures of the respective limb were screened.

Pathohistological screenings revealed lesions in the brain, serosa and spleen in single animals of the placebo group. Scoring of fibrinosuppurative lesions of challenged piglets did not reveal substantial differences between the two groups (Additional file 6). In summary, the challenge experiment did not reveal any protection against disease induced through *S. suis cps14* challenge.

### *S. suis*-induced oxidative burst as a parameter to estimate protection against invasive infection with *cps14* strain

**Figure 6:**
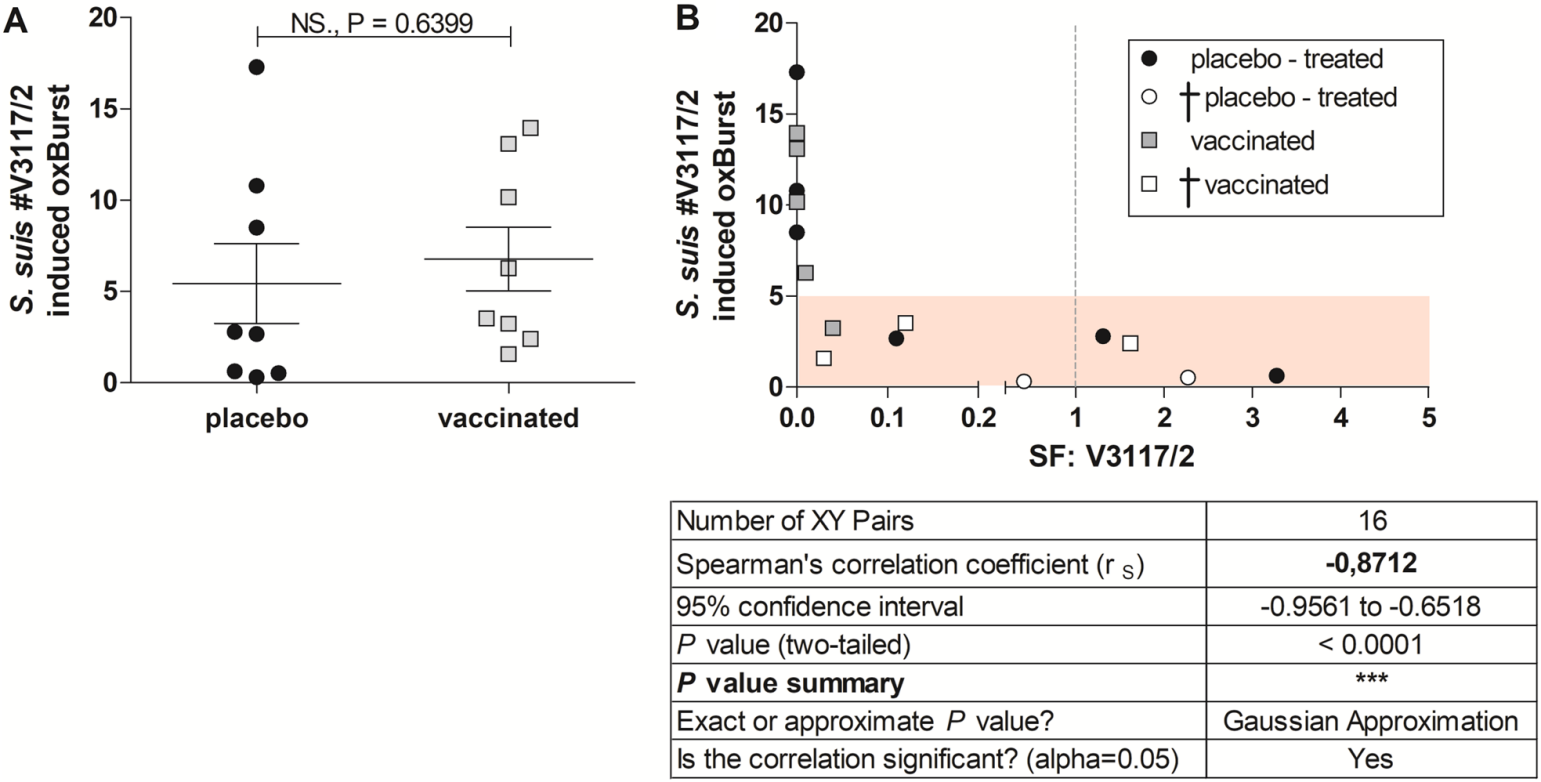
**Oxidative burst of granulocytes is not associated with immunization (A), but with reduced bacterial survival (B).***S. suis* strain V3117/2 (*cps14*)‐induced oxidative burst rates of granulocytes from blood samples drawn 11 d post booster did not reveal an immunization effect of the multicomponent vaccine, but showed correlation with the respective bacterial survival factors (SF). To determine oxidative burst induced by *S. suis* in granulocytes, 6 × 10^6^ CFU/mL V3117/2 or PBS (negative-control) were added to the whole blood samples (11 dp booster) and incubated for 15 min at 37 °C in water bath and for further 10 min after addition of dihydrorhodamin 123 (5 μg/mL). Following erythrocyte lysis the samples were directly measured by flow cytometry. *S. suis*-induced oxidative burst rates were calculated as described in “Materials and methods”. **A** Comparison of *S. suis*-induced oxidative burst rates of placebo-treated (black circle) and vaccinated (grey square) animals. Statistical analysis was conducted with the unpaired t-test (two-tailed). **B** Spearman correlation between *S. suis* induced-oxidative burst rates with the respective bacterial survival factors of *S. suis* V3117/2 (*cps14*) in blood of all animals (*n* = 16) as shown in Figure 4. Animals that had to be euthanized after challenge with *S. suis* V3117/2 (*cps14*) due to a high clinical score are marked with white symbols (†*n* = 5).

In a previous study we demonstrated that the *S. suis* induced production of ROS in granulocytes is antibody-mediated and leads to a reduced bacterial survival in blood [26]. To illuminate the role of *S. suis*-induced oxidative burst in granulocytes in this immunization-challenge experiment, we determined the induction of oxidative burst by the challenge *S. suis* strain V3117/2 in blood samples drawn 11d after booster immunization. We detected a wide range of *S. suis*-induced burst rates between 0 and 17% by the *cps14* strain V3117/2. Between placebo-treated and vaccinated animals we found no statistical difference (Figure 6A). To analyze, whether the induction of oxidative burst in blood granulocytes before challenge is related to protection, the *cps14* challenged animals were divided in a convalescent (filled symbols in Figure 6B) and a susceptible subgroup for cases with euthanasia based on a high clinical score (open symbols in Figure 6B). The oxidative burst rate shows a strong negative correlation with bacterial survival factors (Spearman r_S_ = −0.87 with *p* < 0.0001; Figure 6B). Notably, animals with *S. suis*-induced oxidative burst < 5% (*n* = 9) show a clearly reduced survival (4/9), whereas all animals with an *S. suis*-induced oxidative burst > 5% (*n* = 7) survived the challenge (7/7). These results confirm that induction of oxidative burst in blood granulocytes is important for protection against *S. suis* and can be considered as a correlate of protection. However, it appears unlikely that the antibodies crucial for ROS induction were related to vaccination as we did not observe a difference between vaccinated and placebo-treated piglets.

**Figure 7:**
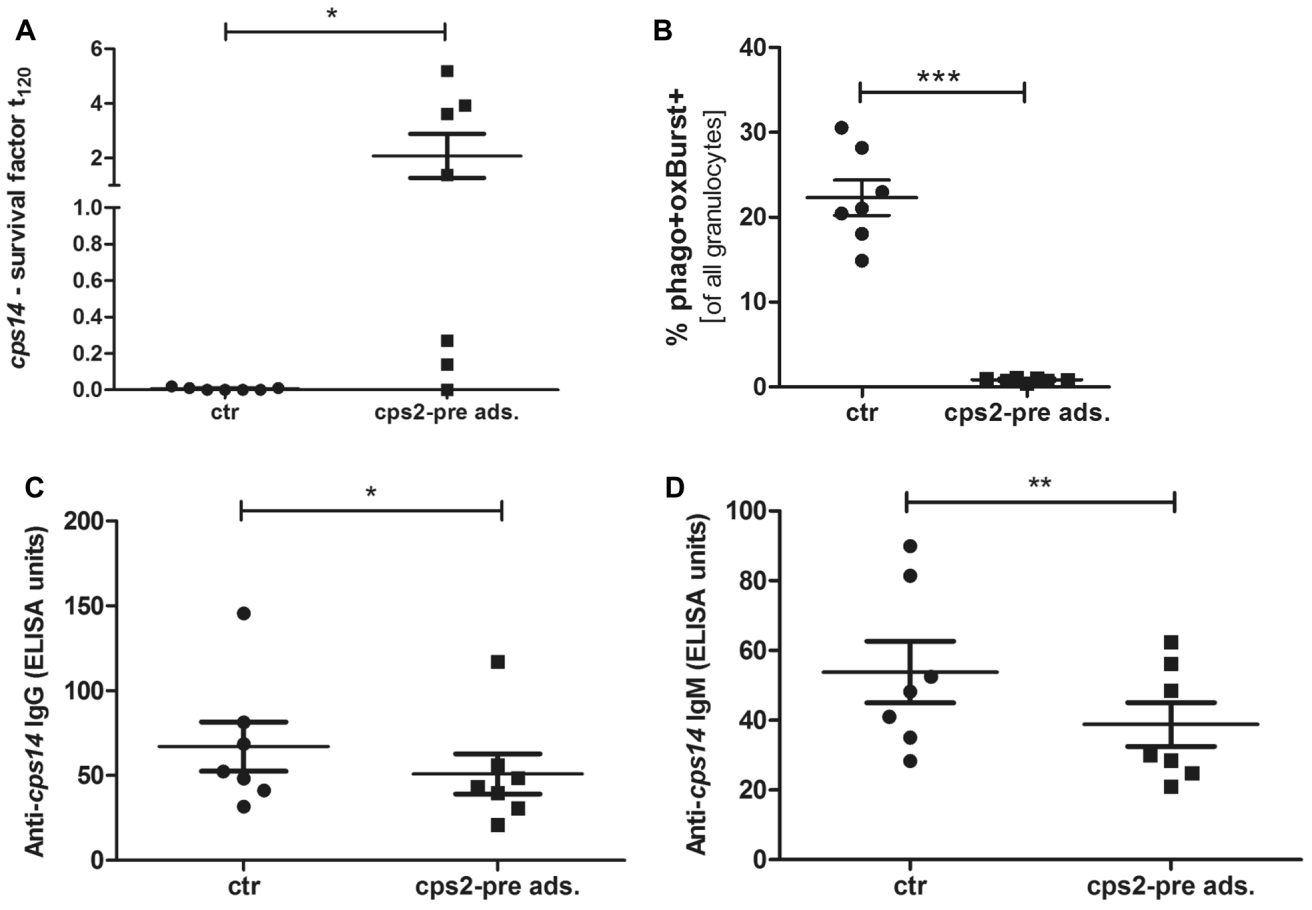
**Pre-adsorption of sera with *cps2* led to increased survival of *cps14* (A) and decreased *cps14*-induced oxidative burst (B).** Sera collected from animals 11 days post booster immunization, which showed an V3117/2‐induced oxidative burst > 5% (*n* = 7), were pre-adsorbed with the *cps2* strain isolated from a diseased piglet not included in the challenge study. Pre-adsorbed and untreated control sera (ctr) were used to reconstitute porcine blood, which was either incubated with 3 × 10^5^ CFU/mL of V3117/2 for bactericidal assay (**A**) or 6 × 10^6^ CFU/mL of Cell Trace^®^ FarRed-labbeld V3117/2 before adding dihydrorhodamine 123. Frequency of granulocytes showing both, oxidative burst and phagocytosis activity induced by *cps14* strain V3117/2 was measured by flow cytometry (BD FACSCalibur and FlowJo) (**B**). Levels of IgG (**C**) and IgM (**D**) antibodies binding to the surface of *cps14* were measured in non-adsorbed (ctr) and pre-adsorbed sera (*cps*2-pre ads.). Mean values are indicated by horizontal lines, standard deviations by error bars. Significant differences between treated and control sera were either determined using Wilcoxon matched pair test (**A** + **C**) or paired t-test (**B** + **D**) (*p < 0.05, **p < 0.01, ***p < 0.001, ****p < 0.0001).

We hypothesized that antibodies elicited through natural infection with a c*ps2* strain present in the original herd might have affected the outcome of the experimental infection. To investigate this, we pre-adsorbed the investigated sera from animals with an *S. suis*-induced ROS > 5% with the c*ps2* strain isolated from the diseased piglet prior to the trial (strain 7119-1). As shown in Figure 7, this led to significantly decreased ROS in granulocytes after phagocytosis of *S. suis* strain V3117/2. Furthermore, we observed an increased bacterial survival factor of V3117/2 in reconstituted blood assay using pre-adsorbed sera compared to untreated sera. In fact, using ELISA we were able to show that the pre-adsorbtion led to a significant decrease of antibodies binding to the surface of V3117/2, whereby the difference between treated and untreated control sera was greater in IgM than in IgG antibody levels. These results suggest that antibodies elicited through natural infection with a c*ps2* strain might have been important for the outcome of the challenge experiment due to cross-reaction with *cps14*.

## Discussion

Sera drawn from piglets in convalescence after *S. suis* infection recognize numerous immunogenic surface-associated proteins. Some of these immunogens are expressed by different serotypes that made these immunogens attractive candidates for cross-protective vaccines in our opinion. In accordance with this reasoning we investigated the protective efficacy of a multicomponent vaccine including six different immunogens expressed by different serotypes (Figures 1 and 2; Additional file 4). Of note, the included lipoprotein SSU1664, also known as oligopeptide-binding protein OppA, was already known to be an immunogenic protein of *cps2* strains [44, 45]. The immunogens selected for the multicomponent vaccine were prominently recognized by convalescence sera and hyperimmune sera, many of which mediated killing of different serotypes in bactericidal assays or opsonophagocytosis assays. This made us even more believe that they are putative protective antigens. Our assumption seemed to be supported by the finding that IgG antibody levels against these immunogens in sera of susceptible piglets were significantly lower. Furthermore, we recorded significantly elevated IgG levels against 5 of these 6 immunogens in sera taken before experimental infection of piglets surviving the following *S. suis* challenge compared to sera of piglets succumbing to the infection (compare pre-infection sera [blue color] and sera of susceptible piglets [green color] in Figure 1). Though vaccinated piglets demonstrate high IgG antibody titers against all six immunogens in contrast to placebo-treated piglets (Figure 3), experimental challenge with *cps14* failed to demonstrate protection. Furthermore, the results of a bactericidal assay also indicate susceptibility to *S. suis cps9* bacteremia in vaccinated piglets as the investigated *cps9* strain proliferated in the blood of these piglets in vitro (survival factor > 1). Accordingly, we discarded the hypothesis that the six selected immunogens are strong protective antigens, at least not against morbidity induced by *cps14* infection (Figure 5) and most likely also not against *cps9* bacteremia (Figure 4). It is also questionable whether other main immunogens of *S. suis* are protective antigens. Muramidase-released protein (MRP) and surface antigen one (SAO) are main immunogens of *S. suis cps2* [13, 46], but piglets with high levels of antibodies against these immunogens developed disease after experimental *cps2* infection in different studies [13, 46–49].

One possible explanation for the ineffectiveness is that these immunogens might not be accessible to antibodies on the surface of the pathogen, at least not during bacteremia. However, our flow cytometric analysis indicates that rabbit hyperimmune sera recognized the selected immunogens on the surface of *S. suis cps*9 strain 16085/3b after cultivation in porcine plasma (Figure 2B). Thus, it appears likely that these immunogens are expressed during bacteremia. However, immunization did not result in significant bactericidal activity of porcine blood. Most strikingly, the *S. suis cps9* strain proliferated in blood of vaccinated piglets in vitro, though this strain shows very prominent binding of immunogen-specific IgG raised in rabbits, especially after cultivation in porcine plasma (Figure 2B). During bacteremia many *S. suis* bacteria are attached to phagocytes without being phagocytosed [50, 51]. This association might be crucial for breaching host barriers and therefore pathogenesis. However, it is unknown if this association with phagocytes has an impact on antibody recognition. A limitation of the phenotypic characterization of *S. suis* by flow cytometry after cultivation in plasma is the absence of phagocytes.

Many immunogens such as MRP are not only present on the bacterial surface but are also released into the environment of the bacterium [15]. Though we confirmed that SSU0934 is surface-located, Gómez-Gascón et al. [52] identified SSU0934 in an immunosecretomic study indicating that this immunogen is also released into the environment. Release of strong immunogens might limit binding of antibodies to the bacterial surface and induction of opsonophagocytosis in vivo. This might constitute an evolutionary advantage for the pathogen and deteriorate the protective efficacy of an immunogen-based vaccine.

The protective efficacy of an *S. suis* vaccine does not only depend on the antigen but also on the adjuvant [47, 49] and the vaccination protocol. Though we cannot rule out that there are better adjuvants for this multicomponent vaccine, it should be noted that we have been using the water-in-oil adjuvant Emulsigen for a *S. suis* bacterin and also for a recombinant *S. suis* vaccine in previous studies using comparable vaccination protocols leading to significant protection against *cps2* [53, 54]. Therefore, it appears unlikely that the adjuvant is the main reason for the disappointing protective efficacy.

One limitation of our and other immunoproteomics studies is that the included convalescence and hyperimmune sera might mediate killing of *S. suis* mainly by capsule-specific antibodies and not by antibodies recognizing protein antigens. Accordingly, some convalescence sera included in our study mediated prominent killing in the opsonophagocytic assay only against the infection strain (e.g. sera of piglets 33 and 150; Additional file 2). Sera of piglets mediating killing of multiple serotypes generally recognize a large set of antigens that makes it very difficult to identify the crucial antigens (results not shown).

In a previous study we demonstrated that ROS induction in *S. suis* infected porcine blood is antibody and complement dependent [26]. In the present study, ROS induction in neutrophils after in vitro *cps14* infection did not exhibit differences between blood drawn from placebo-treated and vaccinated piglets (Figure 6A). However, in porcine blood with high bactericidal activity against *S. suis cps14*, we observed strong induction of ROS (> 5%). As ROS induction was not associated with vaccination, we suppose that antibodies crucial for the high ROS induction were induced through infection with strains colonizing piglets of this herd. Based on thorough screening of *S. suis* isolates of piglets from the original herd over many years we are convinced that this herd is free of *S. suis cps1* and *cps14.* However, piglets of the original herd including the piglets used in this study were colonized with a *cps2 S. suis* strain of sequence type 28 which is not related to clonal complex 1 harboring the challenge strain. This strain caused disease in one piglet originally planned to be included in the challenge experiment. Thus, it appears likely that the other piglets developed a serological response against this strain. Of note, the capsule of *S. suis cps14* and *cps2* share an identical sialic acid-containing side chain [55, 56]. Recognition of *cps2* capsule by rabbit anti-*cps2* serum is significantly reduced when sialic acid is removed [57], suggesting that the antibody response against *cps2* in rabbits is mainly directed against the side chain. Therefore, one might speculate that infection with *S. suis cps2* might induce capsule-specific IgM antibodies cross-reacting with the capsule of *cps14*. However, such cross-reaction has not been shown for porcine sera and serum raised in rabbits against *cps2* as use for agglutination generally does not cross-react with *cps14* suggesting important conformational differences [57]. In the present study, pre-adsorption of sera with the isolated *cps2* strain led to a significant decrease of IgM antibody levels binding to *cps14* (Figure 7) which is in accordance with cross-reacting IgM antibodies. These cross-reacting IgM antibodies might have made a big difference for survival in porcine blood and induction of ROS as IgM is a strong inducer of the classical complement pathway. In fact, we were able to observe increased bacterial survival and reduced *cps14*-induced phagocytosis and oxidative burst after pre-adsorption of sera with the isolated *cps2* strain (Figure 7). It is, however, also possible that antibodies against protein antigens made a difference in ROS induction.

In summary, the results of this study question the idea that strong immunogens expressed by different *S. suis* serotypes are a priori good candidates for protective antigens. *S. suis* colonizes different mucosal surfaces and tonsils during the entire life of a pig. After weaning, most conventional piglets go through an adaptive immune response against *S. suis* [15]. This pig-adapted pathogen has most likely gone through a substantial evolutionary selection against antibody-mediated elimination. Recruitment of host proteins to the bacterial surface, attachment to host cells, formation of biofilms, hiding immunogens under a thick capsule and release of immunogens in the environment are all putative mechanisms that allow this pathogen to survive even in the presence of specific antibodies. Thus, the development of a cross-protective *S. suis* vaccine remains a major challenge.

## Supplementary Information

**Additional file 1:Oligonucleotides used for recombinant expression of *S. suis* antigens in *E. coli***. Name and sequences (5’-3’) of oligonucleotide primers used for construction of expression vectors of recombinant proteins included in the multicomponent vaccine (SSU0934, SSU1869, SSU0757, SSU1950, SSU1664, SSU0187).

**Additional file 2:Origin and bactericidal activity of sera of susceptible piglets, sera pre-infection and sera post-infection**. Information on the original experimental infection and results of bactericidal as well as opsonophagocytosis assays of sera defined as sera of susceptible pigs, sera pre-infection and sera post-infection.

**Additional file 3:** **Origin and activity of hyperimmune sera in opsonophagocytosis assays.**

**Additional file 4:Multiple sequence alignment (tblastn) of antigens in different *Streptococcus suis* strains**. Multiple sequence alignments of each antigen were conducted using sequences retrieved from the genome sequences of *S. suis* strains 10, 13-00283-02 and 16085/3b. As query served the respective sequence of strain P1/7. Dots represent identities, whereas differences are highlighted in red.

**Additional file 5:SDS-PAGE and Western blot analysis of antigens included in the multicomponent vaccine**. The purified proteins were run on SDS–polyacrylamide gel (A), transferred to membranes and probed with antisera raised in rabbits against each antigen (B-G). Numbers on the left are molecular masses in kDa.

**Additional file 6: Scoring of fibrinosuppurative lesions of piglets challenged with *S. suis cps14. ***Fourteen days after prime-booster immunization with multicomponent vaccine or placebo, sixteen growing piglets (*n* = 8 per group) were infected intranasally with 5 × 10^9^ CFU of *S. suis* *cps14* V3117/2. Five vaccinated animals and four placebo animals demonstrated clinical signs of severe disease. In both groups two piglets had to be euthanized for animal welfare reasons because of signs of polyarthritis. One vaccinated animal showed additional signs of central nervous system dysfunction (opisthotonus, generalized tremor, ataxia) and had to be euthanized as well. Surviving piglets were sacrificed fourteen days post-infection. Necropsies and histopathological screenings of the indicated tissues were conducted with all 16 piglets as described previously [10].

## Data Availability

The datasets analyzed during the current study are available from the corresponding author on reasonable request.

## Abbreviations

BSA: Bovine serum albumin
CFU: Colony forming unit
*E.*: *Escherichia*
FITC: Fluorescein isothiocyanate
HRP: Horseradish-peroxidase
LB: Luria–Bertani
MLST: Multi locus sequence typing
MRP: Muramidase-released protein
ORF: Open reading frame
PBS: Phosphate buffered saline
ROS: Reactive oxygen species
SAO: Surface antigen one
*S.*: *Streptococcus*
SDS-Page: Sodium dodecyl sulfate polyacrylamide gel electrophoresis
SF: Survival factor
TBST: Tris-buffered saline with 0.05% Tween 20
THB: Todd Hewitt broth
