# Supplementary material for: Immunogenicity and protective efficacy of a Streptococcus suis vaccine composed of six conserved immunogens
Source: Vet Res. 2021 Aug 25;52:112. doi: 10.1186/s13567-021-00981-3 (PMC8390293; doi:10.1186/s13567-021-00981-3)
Supplement: Supplementary file 1 — Additional file 1:Oligonucleotides used for recombinant expression of S. suis antigens in E. coli. Name and sequences (5’-3’) of oligonucleotide primers used for construction of expression vectors of recombinant proteins included in the multicomponent vaccine (SSU0934, SSU1869, SSU0757, SSU1950, SSU1664, SSU0187). [file 13567_2021_981_MOESM1_ESM.pdf]

Additional file 1: Oligonucleotides used for recombinant expression of *S. suis* antigens in *E. coli*.

Name	Sequence (5'-3')
0934(-21aa)-F	AGGCGGTACCGGTAACCGTGCTTCAAAAAGCA
0934-R	AGGCAAGCTTTTAAGGTTTTTCAGGAACTTCTAC
1869(-20aa)-F	AGGCGAGCTCCGTCCATCGCAAACCACAGAA
1869-R	AGGCGTCGACCTATTTCAGATATTTAACCATCAGT
757Pro-F	AGAGGATCCGATGAATTGACAAGCCTTGTAG
757Pro-R	GGGGTCGACTTAGACTTCTTCTTTATGACG
1950(-30aa)-F	GGCGGTACCGAAACCTATACTGTAAAGTCTGG
1950-R	GGCAAGCTTTTAGTACCAACCATTTGCCAAC
1664(-31aa)-F	GGCGAGCTCGCAAAACAGGAATTGTCATTCC
1664-R	GGCGTCGACTTATTCTGCCACTACACCCTTA
0187-F	GGCGAGCTCATGCGCTTTAATCAATTTTCTTTCA
0187(-stop)-R	GGCAAGCTTGTGCGGTAAAATTAACTGTGATT
pQE80L-strep-F	AGCGCGTGGAGCCATCCGCAGTTTGAAAAATAAAATTAGCTGAGCTTGGACTCCT
pQE80L-HindIII-R	AAGCTTGGCTGCAGGTCGAC
