# Supplementary material for: Immunogenicity and protective efficacy of a Streptococcus suis vaccine composed of six conserved immunogens
Source: Vet Res. 2021 Aug 25;52:112. doi: 10.1186/s13567-021-00981-3 (PMC8390293; doi:10.1186/s13567-021-00981-3)
Supplement: Supplementary file 2 — Additional file 2:Origin and bactericidal activity of sera of susceptible piglets, sera pre-infection and sera post-infection. Information on the original experimental infection and results of bactericidal as well as opsonophagocytosis assays of sera defined as sera of susceptible pigs, sera pre-infection and sera post-infection. [file 13567_2021_981_MOESM2_ESM.pdf]

Additional file 2: Origin and bactericidal activity of sera of susceptible piglets, sera pre-infection and sera post-infection

Group of sera	Serum	Challenge strain	Sides of detection (challenge strain)					Bacterial survival factor[a] (- not investigated)						
								Bactericidal Assay				Opsonophagocytosis Assay		
			Serosa	Spleen/ Liver	Brain/ CSF[b]	Joint fluid	Endocard	*cps14*	*cps2*	*cps7*	*cps9*	*cps2*	*cps7*	*cps9*
Sera of susceptible piglets	4546	*cps14 (V3117/2)*	x	x		x		60.3	35.5	-	0.9	-	-	-
	5732	*cps14 (V3117/2)*		x	x	x	x	2.8	1.9	-	0.9	-	-	-
	5831	*cps14 (V3117/2)*				x		26.5	6.1	-	1.2	-	-	-
	9970	*cps2 (10)*	x	x	x		x	-	-	-	-	-	-	-
	9747	*cps2 (10)*	x	x		x	x	-	-	-	-	-	-	-
	9777	*cps2 (10)*		x	x	x	x	-	-	-	-	-	-	-
	9884	*cps2 (10)*		x	x	x		-	-	-	-	-	-	-
	9807	*cps2 (10)*		x	x			-	-	-	-	-	-	-
	2886	*cps2 (10)*		x	x	x		-	6.6	-	-	-	-	-
	2843	*cps2 (10)*		x	x		x	-	8.5	-	-	2.0	14.4	0.9
	2858	*cps2 (10)*	x	x				-	8.6	-	-	2.4	12.9	1.7
	3	*cps7 (13-00283-02)*		x	x	x		-	0.1	44.8	0.2	-	-	-
	6	*cps7 (13-00283-02)*				x		-	0.4	54.9	0.3	-	-	-
	12	*cps7 (13-00283-02)*	x	x		x		-	0.4	62.3	1.1	-	-	-
	36	*cps7 (13-00283-02)*				x		-	0.0	67.1	0.4	0.1	10.7	0.5
	15	*cps9 (16085/3b)*		x	x	x	x	-	3.1	-	3.2	-	-	-
	28	*cps9 (16085/3b)*	x	x	x	x	x	-	4.0	-	1.5	0.0	12.8	0.5
	30	*cps9 (16085/3b)*	x	x	x			-	6.7	-	1.6	-	-	-
	39	*cps9 (16085/3b)*			x				0.3	-	0	0.1	10.8	0.3
	8882	*cps9 (A3286/94)*			x			-	3.6	-	2.4	-	-	-

Sera group	Serum	Challenge strain	Sides of detection (challenge strain)					Bacterial survival factor[a]						
								Bactericidal Assay				Opsonophagocytosis		
			Serosa	Spleen/ Liver	Brain/ CSF[b]	Joint fluid	Endocard	*cps14*	*cps2*	*cps7*	*cps9*	*cps2*	*cps7*	*cps9*
Sera pre-infection	5649	*cps14 (V3117/2)*						0.00	1.4	-	0.2	-	-	-
	5663	*cps14 (V3117/2)*						0.00	0.0	-	1.3	-	-	-
	1	*cps2 (10)*						-	3.7	-	-	-	-	-
	33	*cps2 (10)*						-	0.9	-	-	-	-	-
	107	*cps2 (10)*						-	-	-	-	-	-	-
	150	*cps2 (10)*						-	-	-	-	-	-	-
	9898	*cps2 (10)*						-	-	-	-	-	-	-
	9506	*cps2 (10)*						-	-	-	-	-	-	-
	9725	*cps2 (10)*						-	-	-	-	-	-	-
	2786	*cps2 (10)*						-	0.0	0.0	-	-	-	-
	3651	*cps2 (10)*						-	8.2	-	-	2.1	11.6	1.1
	3561	*cps2 (10)*						-	0.0	-	-	0.2	10.9	1.4
	9	*cps7 (13-00283-02)*						-	0.7	25.4	0.5	-	-	-
	17	*cps7 (13-00283-02)*						-	0.0	40.1	0.6	-	-	-
	24	*cps7 (13-00283-02)*						-	0.8	47.5	0.9	-	-	-
	43	*cps7 (13-00283-02)*						-	0	11.9	0.5	0.3	16.0	0.3
	4403	*cps7 (#451)*						-	2.1	11.7	0.1	-	-	-
	132	*cps9 (A3286/94)*						-	-	-	-	-	-	-
	370	*cps9 (8882/3; A3286/94-*						-	0.6	0.0	0.3	-	-	-
	20	*cps9 (16085/3b)*						-	2.4	-	0.0	1.3	12.0	0.3

Sera group	Serum	Challenge strain	Sides of detection (challenge strain)					Sides of detection (challenge strain)						
								Bactericidal Assay				Opsonophagocytosis		
			Serosa	Spleen/ Liver	Brain/ CSF[b]	Joint fluid	Endocard	*cps14*	*cps2*	*cps7*	*cps9*	*cps2*	*cps7*	*cps9*
Sera post-infection	5649	*cps14 (V3117/2)*						-	-	-	-	0.6	0.1	1.0
	5663	*cps14 (V3117/2)*						-	-	-	-	0.1	0.0	1.3
	1	*cps2 (10)*						-	-	-	-	0.0	0.5	0.9
	33	*cps2 (10)*						-	-	-	-	0.2	4.7	2.8
	107	*cps2 (10)*						-	-	-	-	2.2	11	3.2
	150	*cps2 (10)*						-	-	-	-	0.2	6.0	4.2
	9898	*cps2 (10)*						-	-	-	-	0.0	0.0	0.5
	9506	*cps2 (10)*						-	-	-	-	0.2	0.0	0.0
	9725	*cps2 (10)*						-	-	-	-	0.1	0.0	0.4
	2786	*cps2 (10)*						-	-	-	-	0.0	0.0	0.3
	3651	*cps2 (10)*						-	-	-	-	0.0	7.0	0.2
	3561	*cps2 (10)*						-	-	-	-	0.0	0.0	0.0
	9	*cps7 (13-00283-02)*						-	0.8	-	0.3	0.3	0.0	0.6
	17	*cps7 (13-00283-02)*						-	1.0	-	4.2	0.8	0.0	1.5
	24	*cps7 (13-00283-02)*						-	0.1	-	0.0	0.8	0.0	0.4
	43	*cps7 (13-00283-02)*						-	-	-	-	2.8	1.8	0.2
	4403	*cps7 (13-00283-02)*						-	0.1	-	-	0.7	0.0	2.2
	132	*cps9 (A3286/94)*						-	-	-	-	3.8	5.7	5.2
	370	*cps9 (8882/3; A3286/94-*						-	-	-	-	0.5	0.2	0.0
	20	*cps9 (16085/3b)*						-	2.5	-	0.0	1.9	0.0	0.0

[a] Bactericidal assays were conducted with heparinized blood drawn from the same animal as the serum sample. The survival factors of bactericidal and opsonophagocytosis assays represented the ratio of CFU at 120 min and 60 min, respectively, to CFU at time zero. A minus (-) indicates that the assay was not conducted.

[b] CSF = Cerebrospinal fluid
