# Supplementary material for: Immunogenicity and protective efficacy of a Streptococcus suis vaccine composed of six conserved immunogens
Source: Vet Res. 2021 Aug 25;52:112. doi: 10.1186/s13567-021-00981-3 (PMC8390293; doi:10.1186/s13567-021-00981-3)
Supplement: Supplementary file 3 — Additional file 3: Origin and activity of hyperimmune sera in opsonophagocytosis assays. [file 13567_2021_981_MOESM3_ESM.pdf]

Additional file 3: **Origin and activity of hyperimmune sera in opsonophagocytosis assays**

Serum	Bacterin strain	Opsonophagocytosis assays (bacterial survival factor; - not investigated)		
		cps2	*cps7*	*cps9*
4515	*cps2 (10)*	0	1.3	0.6
4641	*cps2 (10)*	0.8	0.5	0.0
7013	*cps2 (I9841/1)*	0.9	0.1	0.8
7007	*cps2 (I9841/1)*	0.2	0.0	1.1
33/40	*cps2*	0.1	-	0.4
34	*cps2*	0.2	-	0.2
38	*cps2*	0.4	-	0.0**
39	*cps2*	0.1	-	0.0**
8946	*cps7 (13-00283-02)*	0.0	0.0	1.2
2223	*cps7 (13-00283-02)*	0.1	0.0	0.0
7108	*cps9 (A5863/93)*	1.9	0.0	0.0
7109	*cps9 (A5863/93)*	0.9	0.0	0.1
31	*cps9*	0.2	-	0.6*
35	*cps9*	0.1	-	0.1*
36	*cps9*	1.3	-	0.6*
37	*cps9*	0.8	-	0.8*

* serum was diluted 1:6 for the opsonophagocytosis assay
** serum was diluted 1:2 for the opsonophagocytosis assay
- : not determined
