# Supplementary material for: Immunogenicity and protective efficacy of a Streptococcus suis vaccine composed of six conserved immunogens
Source: Vet Res. 2021 Aug 25;52:112. doi: 10.1186/s13567-021-00981-3 (PMC8390293; doi:10.1186/s13567-021-00981-3)
Supplement: Supplementary file 4 — Additional file 4:Multiple sequence alignment (tblastn) of antigens in different Streptococcus suis strains. Multiple sequence alignments of each antigen were conducted using sequences retrieved from the genome sequences of S. suis strains 10, 13-00283-02 and 16085/3b. As query served the respective sequence of strain P1/7. Dots represent identities, whereas differences are highlighted in red. [file 13567_2021_981_MOESM4_ESM.pdf]

Additional file 4:

Multiple sequence alignment (tblastn) of antigens in different *Streptococcus suis* strains.

SSU0934

```
Program: TBLASTN
Query: SSU0934 ID: lcl|Query_56127(amino acid) Length: 355

Alignments:

Strain P1/7        1        MNKKlVGLGLAAAAAVSLAACGNRASKSSSSEASSSADTSVKAAIVTDIGGVDDRSFNQS  60
Strain 10          962634   ............................................................  962455
Strain 16085/3b    1117036  ............................................................  1117215
Strain 13-00283-02 1177923  ............V...............................................  1177744

Strain P1/7        61       AWEGLQEWGKAASLSKGNGYDYFQSASESDYITNLDSAVAGGYNLVFGIGFALESAIAEV  120
Strain 10          962454   ............................................................  962275
Strain 16085/3b    1117216  ............................................................  1117395
Strain 13-00283-02 1177743  ............................................................  1177564

Strain P1/7        121      APNNPNTHYVIVDSVVPDQDNVVSVGFADHEASYLAGVAAAKSTKTNHVGFIGGMEGVII  180
Strain 10          962274   ............................................................  962095
Strain 16085/3b    1117396  ........................................................D...  1117575
Strain 13-00283-02 1177563  .S..........................................................  1177384

Strain P1/7        181      DRFEAGFVAGAKSVNKDIKITVDYAGSFGDAAKGQTLAAAQYAAGADVIFHASGGTGNGV  240
Strain 10          962094   ............................................................  961915
Strain 16085/3b    1117576  ............................................................  1117755
Strain 13-00283-02 1177383  ............................................................  1177204

Strain P1/7        241      FAAAKAENETRNEADKVWVIGVDRDQSAEGTYTSKDGKSSNFVLASTLKQVGTSVKDIAT  300
Strain 10          961914   ............................................................  961735
Strain 16085/3b    1117756  ............................................................  1117935
Strain 13-00283-02 1177203  ............................................................  1177024

Strain P1/7        301      KAAAGDFPGGQVLQFSLKDKGVELAETNLSEDASKAVADAKQAILDGKVEVPEKP  355
Strain 10          961734   .......................................................  961570
Strain 16085/3b    1117936  .......................................................  1118100
Strain 13-00283-02 1177023  .......................................................  1176859
```

SSU1869

```
Program: TBLASTN
Query: SSU1869 ID: lcl|Query_15367(amino acid) Length: 307

Alignments:

Strain P1/7        20       CRPSQTTEGSSKPRVAVTTSFLNDMVYQLAGDEVERDLLIPAGEDPHLYVAKSSDLSKLQ  79
Strain 10          1937596  ............................................................  1937417
Strain 16085/3b    2088264  ............................................................  2088085
Strain 13-00283-02 133547   ............................................................  133726

Strain P1/7        80       KADLVLYHGLHFEGKMVEALEKTGVAVSKNFNAKDLNTMDEDGEEIVDPHFWFSIPLYKS  139
Strain 10          1937416  ............................................................  1937237
Strain 16085/3b    2088084  ............................................................  2087905
Strain 13-00283-02 133727   ................................G...........................  133906

Strain P1/7        140      AVAVASEELQKLLPAKAEMIQKNTEKYQAQLDDLHAWVEKELSVIPKESRYLVTPHDAFN  199
Strain 10          1937236  ............................................................  1937057
Strain 16085/3b    2087904  .......................A....................................  2087725
Strain 13-00283-02 133907   .......................A....................................  134086

Strain P1/7        200      YFAASYDFTLYAPQGVSTDSEVANSDMIETVNLIIDHNIKAIFTESTTNPERMKKLQEAV  259
Strain 10          1937056  ............................................................  1936877
Strain 16085/3b    2087724  ............................................................  2087545
Strain 13-00283-02 134087   ............................................................  134266

Strain P1/7        260      KAKGGQVEVVTGEGKELFSDSLAPEGEEGDTFIDMYKHNVKLMVKYLK  307
Strain 10          1936876  ................................................  1936733
Strain 16085/3b    2087544  ................................................  2087401
Strain 13-00283-02 134267   ................................................  134410
```

SSU1664

```
Program: TBLASTN
Query: SSU1664 ID: lcl|Query_15113(amino acid) Length: 596

Alignments:

Strain P1/7        1        MKKTTKLFALAGVTLLSASVLAACGSKQSGAAKQELSFPAEVKQDGTAVADAQLKYAWVS  60
Strain 10          1714321  ............................................................  1714142
Strain 16085/3b    449281   .................................................S..........  449460
Strain 13-00283-02 1827097  .................................................S..........  1826918

Strain P1/7        61       PTTSSGLLIDELTENTTDSTFGGMVDISMFGYDGERKLDDSGLAKAEFDVKGKKITVSLT  120
Strain 10          1714141  ............................................................  1713962
Strain 16085/3b    449461   .......................F................................I...  449640
Strain 13-00283-02 1826917  .......................F................................I...  1826738

Strain P1/7        121      GKDYKWSDGESFTINDYIFTIKSMASKDYTGIRFDDKFLNIEGMQEFVDGKASDISGIKK  180
Strain 10          1713961  ............................................................  1713782
Strain 16085/3b    449641   ..........P.................................................  449820
Strain 13-00283-02 1826737  ..........P.................................................  1826558

Strain P1/7        181      VDDYTVELTVKEMSPSMMYAGGDVPAYIQPEHIYKDIPVADWEKSEYSRTAKLVGMGPWK  240
Strain 10          1713781  ............................................................  1713602
Strain 16085/3b    449821   ............................................................  450000
Strain 13-00283-02 1826557  ............................................................  1826378

Strain P1/7        241      IKEIVNGESITYVPNEYFFKGTKPKTSSLKIDIVSPDTIVSEMKAGNYDIAEMPVDQLDS  300
Strain 10          1713601  ............................................................  1713422
Strain 16085/3b    450001   ............................................................  450180
Strain 13-00283-02 1826377  ............................................................  1826198

Strain P1/7        301      YKDASNLNIVGQLESSYEYISFNFGKYDEAAGKNVMDENAKMNDVKLRQAIAYAIDTKTA  360
Strain 10          1713421  ............................................................  1713242
Strain 16085/3b    450181   ...V...................L.......N......K.....................  450360
Strain 13-00283-02 1826197  ...V...................L.......N......K.....................  1826018

Strain P1/7        361      GKSLYNGLYHPAKSLIISFFGDIHDSELEGYSYNPEKAKKLLDEAGYKDVDGDGIREGKD  420
Strain 10          1713241  ............................................................  1713062
Strain 16085/3b    450361   ............................................................  450540
Strain 13-00283-02 1826017  ............................................................  1825838

Strain P1/7        421      GKAFKITFAARKRTEANEALVQQYIAWWKEVGLNVELYTGRTVEGKSFYNSVQANDAAID  480
Strain 10          1713061  ............................................................  1712882
Strain 16085/3b    450541   ........................................................P...  450720
Strain 13-00283-02 1825837  ........................................................P...  1825658

Strain P1/7        481      MYAGGWSTGYDPNPSGLWGPIAAFNMSRFVSDENTKLLEAISSAESFDDKKNVENYKAWQ  540
Strain 10          1712881  ............................................................  1712702
Strain 16085/3b    450721   ...................SV.......................................  450900
Strain 13-00283-02 1825657  ...................SV.......................................  1825478

Strain P1/7        541      KYAHEQAFAIPTFESEKITAVNKRVKSYDTKYGSASGTGLAYEGIELTADKGVVAE  596
Strain 10          1712701  ........................................................  1712534
Strain 16085/3b    450901   ................S.V.L.....N...N......K.I.L.N............  451068
Strain 13-00283-02 1825477  ................S.V.L.....N...N......K.I.L.N............  1825310
```

SSU0757

```
Program: TBLASTN
Query: SSU0757 ID: lcl|Query_12999(amino acid) Length: 1585

Alignments:

Strain P1/7        1        MKKKETFSLRKYKIGTVSVLLGAVFLFAGAPSVAADELTSLVETKVEATVPDVIVSESAS  60
Strain 10          789590   ............................................................  789769
Strain 13-00283-02 998224   ............................................................  998403
Strain 16085/3b    693316   ...................................................EA.......  693495

Strain P1/7        61       ESPVVEELVDTSVEATPTDVTTTDNVEETLGSEALENITNTEVEATQPAVETPAISEKKV  120
Strain 10          789770   ............................................................  789949
Strain 13-00283-02 998404   ............................................................  998583
Strain 16085/3b    693496   .........................I..................................  693675

Strain P1/7        121      EEEEKLAVADETTAITNQEEAKPQNIDSNTIITVPKVWDSGYKGEGTVVAIIDSGLDVDH  180
Strain 10          789950   ............................................................  790129
Strain 13-00283-02 998584   ..D.........................................................  998763
Strain 16085/3b    693676   ......SI.......S............................................  693855

Strain P1/7        181      DVLHISDLSTAKYKSEKEIEAAKEAAGITYGEWFNDKVVFGYNYVDVNTVLKEEDKRSHG  240
Strain 10          790130   ............................................................  790309
Strain 13-00283-02 998764   ......................----..................................  998931
Strain 16085/3b    693856   ............................S...............................  694035

Strain P1/7        241      MHVTSIATGNPTQPVAGQLMYGVAPEAQVMFMRVFSDLKATTGAALYVKAIEDAVKLGAD  300
Strain 10          790310   ............................................................  790489
Strain 13-00283-02 998932   ................E...........................................  999111
Strain 16085/3b    694036   ............................................................  694215

Strain P1/7        301      SINLSLGGANGSVVNMNENVTAAIEAARRAGVSVVIAAGNDGTFGSGHSNPSADYPDYGL  360
Strain 10          790490   ............................................................  790669
Strain 13-00283-02 999112   ............................................................  999291
Strain 16085/3b    694216   ............................................................  694395

Strain P1/7        361      VGAPSTARDAISVASYNNTTVGSKVINIIGLENNADLNYGKSSFDNPEKSPVSFEIGKEY  420
Strain 10          790670   ............................................................  790849
Strain 13-00283-02 999292   ..T...............................................S.P.......  999471
Strain 16085/3b    695366                           I...........................P.......  695473
Strain 16085/3b    694396   ........................                                      694470

Strain P1/7        421      EYVYAGIGQASDFDGLNLIGKLALIKRGTISFSEKIANATaagavgvvIFNSRPGEANVS  480
Strain 10          790850   ............................................................  791029
Strain 13-00283-02 999472   ..................T.........................................  999651
Strain 16085/3b    695474   ................D.T...........T.............................  695653

Strain P1/7        481      MQLDDTAIAIPSIFIPLEFGEALASNSYKIAFNNETDIRPNPEAGLLSDFSSWGLSADGE  540
Strain 10          791030   ............................................................  791209
Strain 13-00283-02 999652   ........................AKP...............K.................  999831
Strain 16085/3b    695654   ............V...........T...................................  695833

Strain P1/7        541      LKPDLAAPGGAIYAAINDNDYANMQGTSMASPHVAGAAVLVKQYLQATYPTKSPQEIEAL  600
Strain 10          791210   ............................................................  791389
Strain 13-00283-02 999832   ............................................................  1000011
Strain 16085/3b    695834   ............................................................  696013

Strain P1/7        601      VKHLLMSTAKAHVNKETTAYTSPRQQGAGIIDTAAAISTGLYLTGEDGYGSITLGNVEDI  660
Strain 10          791390   ............................................................  791569
Strain 13-00283-02 1000012  ......................S....................................T  1000191
Strain 16085/3b    696014   ...........................S...............................T  696193

Strain P1/7        661      FSFTVTLHNITNEDKTLNYSTQLTTDTVQNGLITLAPCLLAEIPGGKVTVKANSSTTVTI  720
Strain 10          791570   ............................................................  791749
Strain 13-00283-02 1000192  .....K...............................R......................  1000371
Strain 16085/3b    696194   .....................................R......................  696373

Strain P1/7        721      NVDAASFAEELTGLMKNGYYLEGFVRFTDVADVGDIVSIPYVGFRGEFQNLAVLEEPIYN  780
Strain 10          791750   ............................................................  791929
Strain 13-00283-02 1000372  ....S...........................G...........................  1000551
Strain 16085/3b    696374   ....S...........................G...............P...........  696553

Strain P1/7        781      LIADGKGGFYFEPVTAQPDTVDISHHYTGLVTGSTELIYSTDKRSDFAIKTLGTFKNEAG  840
Strain 10          791930   ............................................................  792109
Strain 13-00283-02 1000552  ............................................................  1000731
Strain 16085/3b    696554   ............................................................  696733

Strain P1/7        841      YFVLELDESGKPHLAISPNGDDNQDSLALKGVFLRNYTDLVASVYAADDTERTNPLWESQ  900
Strain 10          792110   ............................................................  792289
Strain 13-00283-02 1000732  ............................F...............................  1000911
Strain 16085/3b    696734   ............................F...............................  696913

Strain P1/7        901      PQSGNKNFYSGDPKNPKSSIIYPTEWNGTDSEGNALADGKYQYVLTYSSEVPGAAVQTMI  960
Strain 10          792290   ............................................................  792469
Strain 13-00283-02 1000912  ............................................................  1001091
Strain 16085/3b    696914   ............................................................  697093

Strain P1/7        961      FDVIIDRESPVITTATYDETNFTFNPRPAIEKGESGLYREQVFYLVADASGVTTIPSLLE  1020
Strain 10          792470   ............................................................  792649
Strain 13-00283-02 1001092  ...................................................M........  1001271
Strain 16085/3b    697094   ............................................................  697273

Strain P1/7        1021     NGDVTVSDNKVFVAQNDDGSFTLPLDLADISKFYYTVEDYAGNISYEKVENLISIGNEKG  1080
Strain 10          792650   ............................................................  792829
Strain 13-00283-02 1001272  ............................................................  1001451
Strain 16085/3b    697274   ............................................................  697453

Strain P1/7        1081     LVTVNILDKDTNSPVPILFSYSVTDETGKIVAELPRDAGDTSVLKLPFGTYTFDLFLYDT  1140
Strain 10          792830   ............................................................  793009
Strain 13-00283-02 1001452  ....................................Y.......................  1001631
Strain 16085/3b    697454   ....................................Y.......................  697633

Strain P1/7        1141     EWSSLAGETKAVVTISEENSTAEVNFYVTLKDKANLLVDIDALLPSGSTIQLVTADGQTI  1200
Strain 10          793010   ............................................................  793189
Strain 13-00283-02 1001632  ............................................................  1001811
Strain 16085/3b    697634   ............................................................  697813

Strain P1/7        1201     QLPNAKYSKTDYGKFVPVGTYTILPTLPEGYEFLEELDVAVLANQSNVKKLTLINKVALK  1260
Strain 10          793190   ............................................................  793369
Strain 13-00283-02 1001812  ............................................................  1001991
Strain 16085/3b    697814   ............................................................  697993

Strain P1/7        1261     ELIAELAGLEETARYYNASPELQTAYAKALEDANAVYANKHNQVQVDSALANLVAAREQL  1320
Strain 10          793370   ............................................................  793549
Strain 13-00283-02 1001992  ..........................D................A.....V.S........  1002171
Strain 16085/3b    697994   ...................................I.......A.......S........  698173

Strain P1/7        1321     NGQATDKEKLIAEVSNYTPTQANFIYYNAENTKQIAYDTAVRSAQLVLNQENVTQAVVNQ  1380
Strain 10          793550   ............................................................  793729
Strain 13-00283-02 1002172  ......T.....................................................  1002351
Strain 16085/3b    698174   ........................................I...................  698353

Strain P1/7        1381     ALADLLAAKANLDGQKTDISALRSAVSVSSVLKATDAKYLNASENVKQAYDQAVEAAKAI  1440
Strain 10          793730   ............................................................  793909
Strain 13-00283-02 1002352  ............................................................  1002531
Strain 16085/3b    698354   ............................................................  698533

Strain P1/7        1441     LADESASQASVDQALAVLTSAQAELNGIATSTNDAKEPANTATDKKDEGTVTPPPIDSEK  1500
Strain 10          793910   ............................................................  794089
Strain 13-00283-02 1002532  .........................D.V................................  1002711
Strain 16085/3b    698534   .........................D...............S..................  698713

Strain P1/7        1501     VDVQAPPVKDTGNSGHVSIGQKPNPQPTLPRPVTLQASLSSPNQEKQVTQLPNTGDNDTR  1560
Strain 10          794090   ............................................................  794269
Strain 13-00283-02 1002712  .................P..........................................  1002891
Strain 16085/3b    698714   .................P.....................................E....  698893

Strain P1/7        1561     YYLVLGVIIGLGTLLVSKRRHKEEV  1585
Strain 10          794270   .........................  794344
Strain 13-00283-02 1002892  .........................  1002966
Strain 16085/3b    698894   .........................  698968
```

SSU1950

```
Program: TBLASTN
Query: SSU1950 ID: lcl|Query_59709(amino acid) Length: 184

Alignments:

Strain P1/7        1        MNITLKKNLKTTIAGLVAGISLLTAGVVSAETYTVKSGDTLSEIAETYNTTVEKLAEQNK  60
Strain 10          2021947  ............................................................  2021768
Strain 13-00283-02 69073    ............................................................  69252
Strain 16085/3b    2151737  ............................................................  2151558

Strain P1/7        61       ITNLDFIHVGQVIELGDVTVKAVESSVEQTQSTSTSTATVTTSTYSSNLSAEDAAAKEWI  120
Strain 10          2021767  ............................................................  2021588
Strain 13-00283-02 69253    ..........................I.................................  69432
Strain 16085/3b    2151557  ............................................................  2151378

Strain P1/7        121      AMKESSGSYDARNGIYIGRYQLTNTYLNGDYSPENQERVADAYVASRYGSWSAAKAFWLA  180
Strain 10          2021587  ............................................................  2021408
Strain 13-00283-02 69433    ............................................................  69612
Strain 16085/3b    2151377  ...........................................................E  2151198

Strain P1/7        181      NGWY  184
Strain 10          2021407  ....  2021396
Strain 13-00283-02 69613    ....  69624
Strain 16085/3b    2151197  ....  2151186
```

SSU0187

```
Program: TBLASTN
Query: SSU0187 ID: lcl|Query_19683(amino acid) Length: 755

Alignments:

Strain P1/7        1        MRFNQFSFIKKETSVYLQELDTLGFQLIPDASSKTNLETFVRKCHFLTANTDFALSNMIA  60
Strain 10          186577   ............................................................  186398
Strain 13-00283-02 2036656  ....................GA......NV..............................  2036835
Strain 16085/3b    198988   ....................GA......N...............................  198809

Strain P1/7        61       EWDTDLLTFFQSDRELTDQIFYQVAFQLLGFVPGMDYTDVMDFVEKSNFPIVYGDIIDNL  120
Strain 10          186397   ............................................................  186218
Strain 13-00283-02 2036836  D.................L...............V...............VI...T....  2037015
Strain 16085/3b    198808   D.E...............L...............V...............VI...T....  198629

Strain P1/7        121      YQLLNTRTKSGNTLIDQLVSDDLIPEDNHYHFFNGKSMATFSTKNLIREVVYVETPVDTA  180
Strain 10          186217   ............................................................  186038
Strain 13-00283-02 2037016  ....A................................L......................  2037195
Strain 16085/3b    198628   ....A................................L......................  198449

Strain P1/7        181      GTGQTDIVKLSILRPHFDGKIPAVITNSPYHQGVNDVASDKALHKMEGELAEKQVGTIQV  240
Strain 10          186037   ............................................................  185858
Strain 13-00283-02 2037196  .......I....................................................  2037375
Strain 16085/3b    198448   .........V.........................E.................PA.....  198269

Strain P1/7        241      KQASITKLDLDQRNLPVSPATEKLGHITSYSLNDYFLARGFASLHVSGVGTLGSTGYMTS  300
Strain 10          185857   ............................................................  185678
Strain 13-00283-02 2037376  .............E.....S........................................  2037555
Strain 16085/3b    198268   S.TK.N.......E..I...................................S.......  198089

Strain P1/7        301      GDYQQVEGYKAVIDWLNGRTKAYTDHTRSLEVKADWANGKVATTGLSYLGTMSNALATTG  360
Strain 10          185677   ............................................................  185498
Strain 13-00283-02 2037556  .............................I..............................  2037735
Strain 16085/3b    198088   ..................H..........I..............................  197909

Strain P1/7        361      VDGLEVIIAEAGISSWYDYYRENGLVTSPGGYPGEDLDSLTALTYSKSLQAGDFLRNKAA  420
Strain 10          185497   ............................................................  185318
Strain 13-00283-02 2037736  ......V.................................................S.E.  2037915
Strain 16085/3b    197908   ......V.................................................S.E.  197729

Strain P1/7        421      YEKGLAAERAALDRTSGDYNQYWHDRNYLLHADRVKCEVVFTHGSQDWNVKPIHVWNMFH  480
Strain 10          185317   ............................................................  185138
Strain 13-00283-02 2037916  .......................Q......Q..K..........................  2038095
Strain 16085/3b    197728   ........................G........K.Q........................  197549

Strain P1/7        481      ALPSHIKKHLFFHNGAHVYMNNWQSIDFRESMNALLSQKLLGYENNYQLPTVIWQDNSGE  540
Strain 10          185137   ............................................................  184958
Strain 13-00283-02 2038096  .....V......................................................  2038275
Strain 16085/3b    197548   ............................................................  197369

Strain P1/7        541      QTWTTLDTFGGENETVLPLGTGSQTVANQYTQEDFERYGKSYSAFHQDLYAGKANQISIE  600
Strain 10          184957   ............................................................  184778
Strain 13-00283-02 2038276  ..............A........H.I....A....D......P..........G..V..D  2038455
Strain 16085/3b    197368   ...........GQ.A..........IS...A....D......P.......T.......M.  197189

Strain P1/7        601      LPVTEGLLLNGQVTLKLRVASSVAKGLLSAQLLDKGNKKRLAPIPAPKARLSLDNGRYHA  660
Strain 10          184777   ............................................................  184598
Strain 13-00283-02 2038456  .....D......................................................  2038635
Strain 16085/3b    197188   .....D..............................S.......................  197009

Strain P1/7        661      QENLVELPYVEMPQRLVTKGFMNLQNRTDLMTVEEVVPGQWMNLTWKLQPTIYQLKKGDV  720
Strain 10          184597   ............................................................  184418
Strain 13-00283-02 2038636  ....L.....D.................................................  2038815
Strain 16085/3b    197008   ....L.....D.....I.....................E...D.................  196829

Strain P1/7        721      LELILYTTDFECTVRDNSQWQIHLDLSQSQLILPH  755
Strain 10          184417   ...................................  184313
Strain 13-00283-02 2038816  ...................................  2038920
Strain 16085/3b    196828   .................................Q.  196724
```

Multiple sequence alignments of each antigen were conducted using sequences retrieved from the genome sequences of *S. suis* strains 10, 13-00283-02 and 16085/3b. As query served the respective sequence of strain P1/7. Dots represent identities, whereas differences are highlighted in red.
