# Supplementary material for: Immunogenicity and protective efficacy of a Streptococcus suis vaccine composed of six conserved immunogens
Source: Vet Res. 2021 Aug 25;52:112. doi: 10.1186/s13567-021-00981-3 (PMC8390293; doi:10.1186/s13567-021-00981-3)
Supplement: Supplementary file 5 — Additional file 5:SDS-PAGE and Western blot analysis of antigens included in the multicomponent vaccine. The purified proteins were run on SDS–polyacrylamide gel (A), transferred to membranes and probed with antisera raised in rabbits against each antigen (B-G). Numbers on the left are molecular masses in kDa. [file 13567_2021_981_MOESM5_ESM.pdf]

Additional file 5:

SDS-PAGE and Western blot analysis of antigens included in the multicomponent vaccine.

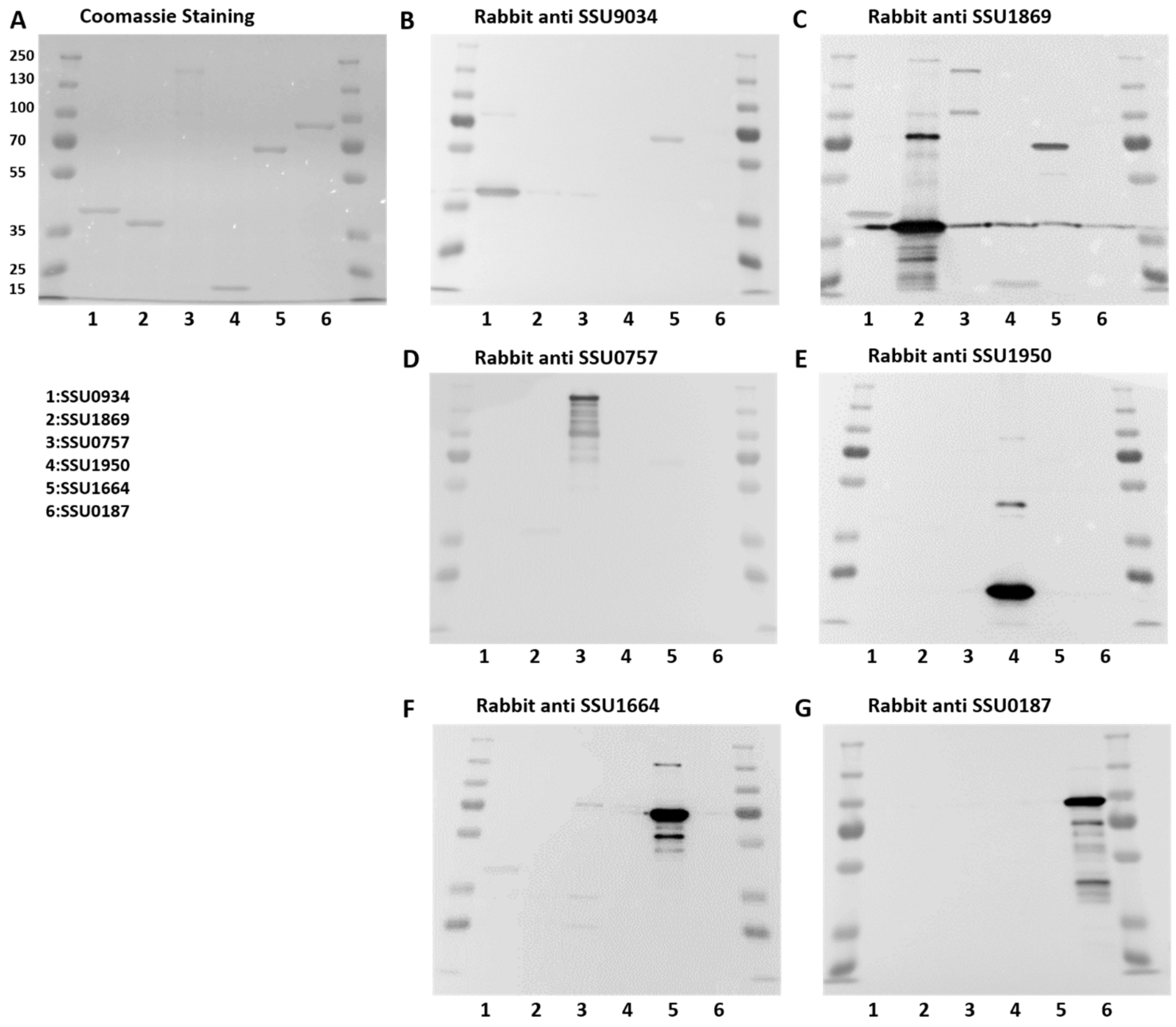

The purified proteins were run on SDS-polyacrylamide gel (A), transfered to membranes and probed with antisera raised in rabbits against each antigen (B-G). Numbers on the left are molecular masses in kDa.
