# Supplementary material for: Immunogenicity and protective efficacy of a Streptococcus suis vaccine composed of six conserved immunogens
Source: Vet Res. 2021 Aug 25;52:112. doi: 10.1186/s13567-021-00981-3 (PMC8390293; doi:10.1186/s13567-021-00981-3)
Supplement: Supplementary file 6 — Additional file 6: Scoring of fibrinosuppurative lesions of piglets challenged with S. suis cps14. Fourteen days after prime-booster immunization with multicomponent vaccine or placebo, sixteen growing piglets (n = 8 per group) were infected intranasally with 5 × 10^9^ CFU of S. suis cps14 V3117/2. Five vaccinated animals and four placebo animals demonstrated clinical signs of severe disease. In both groups two piglets had to be euthanized for animal welfare reasons because of signs of polyarthritis. One vaccinated animal showed additional signs of central nervous system dysfunction (opisthotonus, generalized tremor, ataxia) and had to be euthanized as well. Surviving piglets were sacrificed fourteen days post-infection. Necropsies and histopathological screenings of the indicated tissues were conducted with all 16 piglets as described previously [10]. [file 13567_2021_981_MOESM6_ESM.pdf]

Additional file 6: Scoring of fibrinosuppurative lesions of piglets challenged with *S. suis cps* 14

Immunization	Piglets without lesions[a]	Piglets with lesions in two or more locations[a]	Brain			Serosae			Joint			Spleen and liver			Lung			Heart			
			Meningitis, Chorioiditis			Pleuritis or Peritonitis or Pericarditis			Synovialitis			Splenitis[b] or Hepatitis			Pneumonia			Endocarditis			
			5[c]	3[d]	1[e]	4[c]	2[d]	1[e]	4[c]	2[d]	1[e]	4[c]	2[d]	1[e]	4[c]	2[d]	1[e]	4[c]	2[d]	1[e]	ω[f]
Placebo	6/8	2/8	0/8	1/8	0/8	0/8	1/8	0/8	1/8	0/8	0/8	1/8	1/8	2/8	0/8	1/8	2/8	0/8	0/8	0/8	**1.3**
Vaccine	5/8	3/8	0/8	0/8	0/8	0/8	0/8	0/8	2/8	1/8	0/8	0/8	0/8	0/8	0/8	0/8	1/8	0/8	1/8	1/8	**1.4**

[a] Only fibrinosuppurative lesions are considered. Individual single perivascular neutrophils are not counted.

[b] Neutrophilic accumulation of the splenic red pulp.

[c] Scoring of 4 and 5 indicates moderate to severe diffuse or multifocal fibrinosuppurative inflammations.

[d] Scoring of 2 and 3 indicates mild focal fibrinosuppurative inflammation.

[e] Individual single perivascular neutrophils received a score of 1.

[f] $\omega = \Sigma score_{max}/n_{animals}$
